# Artemether-lumefantrine treatment of uncomplicated *Plasmodium falciparum* malaria: a systematic review and meta-analysis of day 7 lumefantrine concentrations and therapeutic response using individual patient data

**DOI:** 10.1186/s12916-015-0456-7

**Published:** 2015-09-18

**Authors:** WorldWide Antimalarial Resistance Network (WWARN) Lumefantrine PK/PD Study Group

**Affiliations:** WorldWide Antimalarial Resistance Network (WWARN), Oxford, UK; Division of Clinical Pharmacology, Department of Medicine, University of Cape Town, Cape Town, South Africa

**Keywords:** Artemether-lumefantrine, Day 7 lumefantrine concentration, Pharmacokinetic, Pharmacodynamic, Uncomplicated *Plasmodium falciparum* malaria, Baseline parasitemia, Malnutrition, Early parasitological response, Drug resistance, Meta-analysis

## Abstract

**Background:**

Achieving adequate antimalarial drug exposure is essential for curing malaria. Day 7 blood or plasma lumefantrine concentrations provide a simple measure of drug exposure that correlates well with artemether-lumefantrine efficacy. However, the ‘therapeutic’ day 7 lumefantrine concentration threshold needs to be defined better, particularly for important patient and parasite sub-populations.

**Methods:**

The WorldWide Antimalarial Resistance Network (WWARN) conducted a large pooled analysis of individual pharmacokinetic-pharmacodynamic data from patients treated with artemether-lumefantrine for uncomplicated *Plasmodium falciparum* malaria, to define therapeutic day 7 lumefantrine concentrations and identify patient factors that substantially alter these concentrations. A systematic review of PubMed, Embase, Google Scholar, ClinicalTrials.gov and conference proceedings identified all relevant studies. Risk of bias in individual studies was evaluated based on study design, methodology and missing data.

**Results:**

Of 31 studies identified through a systematic review, 26 studies were shared with WWARN and 21 studies with 2,787 patients were included. Recrudescence was associated with low day 7 lumefantrine concentrations (HR 1.59 (95 % CI 1.36 to 1.85) per halving of day 7 concentrations) and high baseline parasitemia (HR 1.87 (95 % CI 1.22 to 2.87) per 10-fold increase). Adjusted for mg/kg dose, day 7 concentrations were lowest in very young children (<3 years), among whom underweight-for-age children had 23 % (95 % CI −1 to 41 %) lower concentrations than adequately nourished children of the same age and 53 % (95 % CI 37 to 65 %) lower concentrations than adults. Day 7 lumefantrine concentrations were 44 % (95 % CI 38 to 49 %) lower following unsupervised treatment. The highest risk of recrudescence was observed in areas of emerging artemisinin resistance and very low transmission intensity. For all other populations studied, day 7 concentrations ≥200 ng/ml were associated with >98 % cure rates (if parasitemia <135,000/μL).

**Conclusions:**

Current artemether-lumefantrine dosing recommendations achieve day 7 lumefantrine concentrations ≥200 ng/ml and high cure rates in most uncomplicated malaria patients. Three groups are at increased risk of treatment failure: very young children (particularly those underweight-for-age); patients with high parasitemias; and patients in very low transmission intensity areas with emerging parasite resistance. In these groups, adherence and treatment response should be monitored closely. Higher, more frequent, or prolonged dosage regimens should now be evaluated in very young children, particularly if malnourished, and in patients with hyperparasitemia.

**Electronic supplementary material:**

The online version of this article (doi:10.1186/s12916-015-0456-7) contains supplementary material, which is available to authorized users.

## Background

The World Health Organization (WHO) recommends artemisinin-based combination therapies (ACTs) for treating uncomplicated *Plasmodium falciparum* malaria [1]. In order to prolong their useful therapeutic life until effective novel antimalarials become available, optimal use and dosing of widely used ACTs is essential. This can only be achieved by accurately defining the therapeutic drug exposure thresholds, which enables identification of vulnerable populations in whom current dosing recommendations do not consistently achieve effective drug exposure. Therapeutic responses are mainly determined by density and susceptibility of the infecting malaria parasites and drug exposure, although acquired host immunity can compensate for failing treatments. For combination therapies, the early parasitological response is determined largely by the artemisinin component. To prevent recrudescence, the malaria parasites that remain after exposure to the artemisinin component for two 48-hr asexual cycles must be cleared by the slowly eliminated partner drug [2]. The precise pharmacokinetic (PK) determinants of treatment outcome in uncomplicated malaria remain uncertain, but the area under the blood or plasma concentration-time curve (AUC) and the concentration on day 7 of slowly eliminated antimalarials are considered important predictors [2, 3].

Artemether-lumefantrine accounted for 73 % of ACTs procured in 2013 [4]. Lumefantrine has variable bioavailability, largely due to fat-dependent absorption, with high plasma protein binding (mainly to high-density lipoproteins) and is extensively metabolized in the liver, primarily by the CYP3A4 enzymes [1]. Lumefantrine concentration on day 7 has been shown to be the most important single concentration measure, in terms of its correlation with the area under the concentration time curve and its association with treatment response [3, 5, 6]. The ‘therapeutic’ day 7 lumefantrine concentrations published to date range from 170 ng/ml to 500 ng/ml [6–12], and were defined mostly from individual studies with small numbers of treatment failures. Lower lumefantrine exposure has been described in young children [9], pregnant women [13–17], smokers [15], or when artemether-lumefantrine is taken unsupervised [9], without fat [18] or with concurrent efavirenz [19–21], rifampicin [22] or mefloquine [23]. However, the extent to which this compromises efficacy is poorly defined, and no dose optimization studies have been published in any of these important target populations.

While there are more studies published on the pharmacokinetics of lumefantrine than any other antimalarial, the individual studies published to date are not sufficient to develop optimal evidence-based dosage recommendations for all major target population groups. The objective of this analysis was to define therapeutic day 7 blood or plasma lumefantrine concentrations for artemether-lumefantrine treatment of uncomplicated *P. falciparum* malaria and to identify patient factors that cause substantial changes to these lumefantrine concentrations. For a drug with an overall high efficacy, this requires a very large sample set, which is most efficiently achieved by pooling available data. In addition to increasing power, using individual patient data allows for standardization of data curation and analysis.

## Methods

### Data acquisition

Relevant studies were identified by searching PubMed, Embase, Google Scholar, ClinicalTrials.gov and conference proceedings using the key words ‘lumefantrine pharmacokinetics’ or ‘lumefantrine concentrations’ and ‘clinical study’. Participating authors agreed to the WorldWide Antimalarial Resistance Network (WWARN) terms of submission [24], which ensure that all data uploaded were anonymized and obtained with informed consent, and in accordance with any laws and ethical approvals applicable in the country of origin. The WWARN automated data management, curation and analysis tools converted submitted data into a set of defined data variables in a standard format, following the WWARN clinical and pharmacology data management and statistical analysis plans [25, 26]. Study reports generated from the formatted datasets were sent back to investigators for validation or clarification.

For the analyses reported here, any study of non-pregnant patients with uncomplicated *P. falciparum* malaria (including mixed infections) treated with a 2- or 3-day artemether-lumefantrine regimen, and with a blood or plasma lumefantrine concentration measurement available on day 7, was eligible for inclusion. Pregnant women were not included as all nine recrudescences in pregnant women were observed in one study in Thailand [16], the only study where lumefantrine concentrations were measured in capillary plasma – precluding disaggregation of the effects of pregnancy and sample matrix on the pharmacokinetic-pharmacodynamic (PK-PD) relationship. The effects of pregnancy on artemether-lumefantrine exposure have been published previously [8, 13–16].

**Fig. 1:**
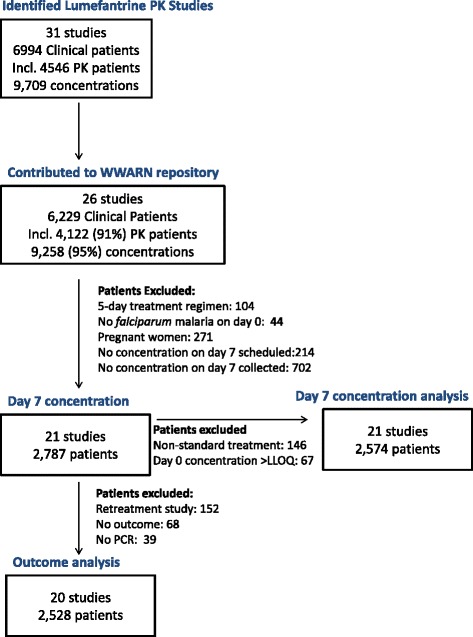
Study profile. PK, pharmacokinetic; LLOQ, Lower limit of quantification

Patients with a quantifiable pre-dose lumefantrine concentration were excluded from the analysis of determinants of day 7 lumefantrine concentration. Studies on re-treatment of treatment failures, or a protocol follow-up period of less than 28 days, or Polymerase Chain Reaction (PCR) results unavailable/indeterminate, were excluded from the outcome analysis (Fig. 1). For the full list of studies [5, 7, 11, 12, 27–44] and assay methods [7, 45–51] used, see Additional file 1: Table S1.

### Ethical approval

All data included in this analysis were obtained after ethical approvals from the countries of origin. Ethical approval to conduct individual participant data meta-analyses was granted by the Oxford Tropical Research Ethics Committee (OxTREC), and OxTREC ruled that appropriate informed consent had been met by each study*.*

### Statistical analysis

All analyses were carried out according to the *a priori* statistical analysis plan [52].

Total dose was calculated from the recorded number of tablets administered per dose for each patient, if this information was available. If no individual patient dosing data was available, dose was estimated using the protocol dosing schedule. All studies in children used weight-based dosing. Treatment was classified as: supervised if all doses had been directly observed; partially supervised if at least the three morning doses had been observed; and not supervised if fewer doses were observed.

A lumefantrine concentration was considered as taken on day 7 if the sample time was recorded as between 144 and 196 hours, inclusive, or as day 6, 7 or 8 after starting artemether-lumefantrine treatment. If multiple concentrations were available within this time interval, the measurement closest to 168 hours was selected. Day 7 concentrations below the lower limit of quantification (LLOQ) were replaced by values half of the limit of quantification [53] (for individual study limits of quantification, see Additional file 1: Table S1). Factors affecting log-transformed lumefantrine concentration on day 7 were investigated using normal regression with random effects for study site in patients treated with the standard artemether-lumefantrine regimen of six doses; those with lumefantrine concentrations > LLOQ before treatment were excluded from this analysis. As treatment supervision is a study-level variable and does not correspond to individual patient compliance with treatment it can mask the effects of other variables, so two multivariable models were fitted: one on all patients, adjusting for whether treatment administration was supervised or not; and another only on patients who received supervised treatment.

Age was analyzed as a categorical variable using <1, 1–4, 5–11 and 12+ years as categories, since pharmacokinetic parameters change with age in children but generally not in adults, and as treatment response improves as premunition is acquired with age in areas of moderate to high malaria transmission intensity. Further categorization in children younger than 5 years of age (1–2 and 3–4 years) was based on heterogeneity of the results; adjusted for dose and other factors, day 7 concentrations were different in these two groups (see Results, Additional file 2: Figure S1, Additional file 3: Figure S2, Table 2).

The hemoglobin cut-offs for moderate anemia were <10 g/dL in children under 5 years of age and <11 g/dL in older patients, and for severe anemia were <7 and <8 g/dL, respectively [54]. The following conversion from hematocrit was used: hemoglobin = (hematocrit-5.62)/2.60 [55].

The nutritional status of children under 5 years of age was calculated as a weight-for-age Z-score (WAZ), using the ‘igrowup’ package developed by WHO [56]. Those with WAZ under −2 (below the 3rd centile) were classified as underweight-for-age (termed ‘underweight’).

Transmission intensity was classified as very low, low, moderate or high, based on triangulation of information given in the original publication(s), observed PCR-confirmed reinfection rates, and the malaria endemicity estimates obtained for study sites and year from the Malaria Atlas Project [57] (Additional file 4: Table S2). Slow early parasitological response was defined as the log_e_ parasite reduction rate at 48 hours (PRR48) <5 (provided a positive count was recorded on day 2), based on the distribution of PRR48 in all patients, or as parasite positivity by microscopy on day 3 [58]. Parasitological response in patients with a negative, or no count, on day 2 and a negative, or no count, on day 3 were classified as unknown.

WHO definitions of therapeutic efficacy outcome [59] were used. Risk factors for PCR-confirmed *P. falciparum* recrudescence and re-infection were examined in two separate analyses. In each analysis, patients with recurrence of *P. falciparum* parasitemia but PCR-confirmed outcome other than the one of interest (for example re-infection in the recrudescence analysis) and patients with a *P. vivax* infection were censored at the time of recurrence. Patients without a PCR genotyping result were excluded to avoid informative censoring. Cox proportional hazards regression was used to model the relationship between treatment outcome and lumefantrine concentration on day 7 and other pre-defined covariates. Random effects in the form of frailty parameters were used to adjust for study site effects [60]. The proportional hazard assumption was tested based on Schoenfeld residuals [61] and, in the case of non-proportionality, interactions with the categorized time variable were used to account for changing effects over time. Fractional polynomials [62] were used to explore possible non-linear forms of continuous variables; the best transformation was linear logarithmic for both lumefantrine concentration and parasitemia.

Pre-defined variables specified in the statistical analysis plan [52] were examined for inclusion in the final models in a stepwise forward fashion. Inclusion of covariates in the final model was based on whether they improved the overall model (likelihood ratio test), changed the coefficient estimates for other factors and examination of the residuals.

Risk of bias within studies was assessed based on: 1) study design (randomization, sequence generation, blinding); 2) methodology for outcome classification (assay methodology and limit of quantification for measurement of lumefantrine concentration on day 7, and PCR methodology for assessing treatment outcome); and 3) the number and proportion of patients with (a) missing outcomes (missing concentration on day 7, incomplete follow-up (<28 days), missing PCR results) and (b) missing baseline covariates (age, weight, parasitemia, temperature, hemoglobin/hematocrit, treatment supervision, dose administration with fat). For the final recrudescence model, two sets of sensitivity analyses were performed. Firstly a model was refitted with each study’s data excluded, one at a time, and a coefficient of variation around the parameter estimates calculated. This would identify any influential studies, that is, studies with unusual results (due to variations in methodology, patient population, and so on) that affect the overall pooled analysis findings. Secondly, to assess the impact of missing PCR data, an unknown outcome was imputed 20 times assuming the same proportion of recrudescent to new infections as observed in patients with known outcome; the model was refitted each time and imputation estimates and their 95 % CI were calculated [63].

## Results

### Data summary

WWARN received and curated data from 26 artemether-lumefantrine pharmacokinetic studies of 6,229 malaria patients in 12 countries in Africa and Asia (Fig. 1, Additional file 1: Table S1). In the initial search carried out in December 2012, a further four studies were identified [10, 64–66] and investigators invited to participate, but their data was not deposited in the WWARN repository so could not be included (one study was contributed after the analysis was completed). The last bibliographic search carried out in December 2014 identified another recent relevant study [13], when it was too late to include in the analysis.

Five studies were excluded as they were studies of pregnant women, did not collect day 7 pharmacokinetic samples or evaluated a five-day artemether-lumefantrine regimen (Fig. 1). Among the 21 artemether-lumefantrine therapeutic efficacy studies included in this analysis, lumefantrine concentrations on day 7 were available for 2,787 patients (Fig. 1, Additional file 1: Table S1), comprising: 82 infants (median 8 months); 1,188 children aged 1–4 years (median 3 years); 564 children aged 5–11 years (median 8 years); and 953 non-pregnant patients aged ≥12 years termed ‘adults’ (range = 12–87 years, IQR = 16 to 30, with 99 % <60 years of age). Among the children aged <5 years, 220/1,269 (17 %) were underweight-for-age (termed ‘underweight’), with a median WAZ of −2.6 (range = −5.3 to −2.0; IQR = −3.1 to −2.27). There were no important clinical differences in baseline characteristics between patients with pharmacokinetic data (from 3 % to 100 % across studies) and the patients in these studies in whom day 7 lumefantrine concentrations were not measured (Table 1). Almost all patients had been treated with Novartis Coartem® tablets (2,756/2,787; 99 %), with very few (31/2,787; 1 %) given Novartis Coartem® Dispersible tablets; none had been treated with a generic artemether-lumefantrine formulation.

**Table 1:** Baseline characteristics of patients^a^ with and without day 7 lumefantrine concentrations

Parameter	Patients with day 7 lumefantrine concentration		Patients without day 7 lumefantrine concentration		*P* value^b^
	N	Median (range) or N (%)	N	Median (range) or N (%)	
Age (years)	2,786	6.2 (0.3 to 86.7)	2,805	5.5 (0.2 to 80.0)	0.028
Age category					<0.001
< 1 year		82 (3.0)		107 (3.8)	
1–2 years		573 (20.6)		590 (21.0)	
3–4 years		615 (22.1)		604 (21.5)	
5–11 years		564 (20.2)		852 (30.4)	
≥ 12 years		953 (34.2)		652 (23.2)	
Weight (kg)	2,786	18.5 (5.0 to 150.0)	2,797	17.0 (5.0 to 97.0)	0.104
Underweight^c^	1,269	220 (17.3)	1,299	218 (16.8)	0.211
Total dose (mg/kg)	2,764	65.5 (19.2 to 144.0)	2,786	65.5 (20.9 to 144)	0.092
Treatment supervision	2,787		2,788		<0.001
Fully		1,971 (70.7)		1,644 (59.0)	
Partially		115 (4.1)		602 (21.6)	
Unsupervised		701 (25.2)		542 (19.4)	
Co-administration with fat	2,787		2,809		0.147
Yes/advised		2,204 (79.1)		1,752 (62.4)	
Not stated		583 (20.9)		1,057 (37.6)	
Parasitemia (/μl)	2,767	17,140 (16 to 524,414)	2,799	18,120 (10 to 862,400)	0.021
Temperature (°C)	2,662	37.7 (34.3 to 41.9)	1,967	37.5 (35.0 to 41.5)	<0.001
Hemoglobin (g/dL)	2,068	10.6 (4.8 to 25)	1,484	11.3 (4.2 to 17.9)	0.428
Sex (female)	2,787	1,281 (46.0)	2,805	1,278 (45.6)	0.857
Gametocytes present	2,418	112 (4.9)	1,654	92 (5.6)	0.797
Moderate anemia	2,507	1,132 (45.1)	1,484	516 (34.8)	0.016
Severe anemia	2,507	155 (6.2)	1,484	57 (3.8)	0.056

^a^Patients enrolled in the 21 studies included in the pooled analysis; ^b^adjusted for study site in a random effects model; ^c^defined using a weight-for-age Z-score (WAZ) < −2 in children <5 years of age

### Dosage regimen

The majority of patients (2,641/2,787; 95 %) were treated with the current WHO recommended six-dose artemether-lumefantrine regimen administered over 3 days (60 hours). The median (range) of the total body weight-adjusted dose received was: 90 (45–144) mg/kg for infants; 65 (38–111) mg/kg for children aged 1–4 years; 72 (48–111) mg/kg for children aged 5–11 years; and 58 (19–108) mg/kg for patients aged 12 years or older (Additional file 2: Figure S1). Underweight children aged 1 to 2 years received higher mg/kg doses than better nourished children of the same age (80 (38–111) mg/kg compared to 65 (38–97) mg/kg; *P* <0.001). However, the opposite was seen in 4-year-old children (60 (45–90) mg/kg compared to 82 (48–111) mg/kg; *P* <0.001), as the better nourished 4-year-olds usually received the two-tablet dose recommended for the weight band 15–24 kg, while underweight 4-year-olds only received the one-tablet dose recommended for the 5–14 kg weight band, as all underweight children weighed <14 kg (Additional file 2: Figure S1). Two alternative regimens (termed ‘non-standard treatment’) were also investigated in adults; in these studies the total recommended dose was given as single daily doses for 3 days (n = 19) or 2/3 of the recommended dose was given as four doses over 2 days (n = 127).

### Day 7 lumefantrine concentrations

Day 7 lumefantrine concentrations were measured either in venous plasma (n = 1,395 (50 %); 16 studies), capillary whole blood dried on filter paper (n = 848 (29 %); 5 studies) or venous whole blood dried on filter paper (n = 544 (19 %); 2 studies) (Additional file 1: Table S1, Additional file 3: Figure S2, Additional file 5: Figure S3). The coefficient of variation of the day 7 lumefantrine concentration (on log scale) by study, after adjusting for mg/kg dose administered, was highest in capillary blood on filter paper ranging between 43–240 %, compared to 24–27 % in venous blood on filter paper and 23–70 % in venous plasma. Hemoglobin was only associated independently with lumefantrine concentrations measured in capillary whole blood samples. For blood collected on filter paper, most assays (97 %) had LLOQ ≥25 ng/ml, while measurements in venous plasma were more sensitive (LLOQ ≤5 ng/ml for 56 % of samples). For lumefantrine concentrations measured in capillary blood on filter paper, 8 % were below the limit of quantification (BLQ), while only 1 % of those in venous plasma or in venous blood on filter paper were BLQ. The majority of children under 5 years of age (59 %) had lumefantrine concentrations measured in capillary blood, while these were measured in venous plasma for the majority of older patients (60 %). Additional file 5: Figure S3 shows the distribution of measured concentrations by study.

The exact time of the lumefantrine concentration sample collection was available in 384 (14 %) patients, with a median of 166 (IQR = 164 to 168) hours. The protocol time in hours was known in 33 (1 %) patients, while in the remaining patients only the day of measurement was recorded. Pre-dose lumefantrine concentrations were measured in 676/2,787 (24.3 %) patients in five studies; these were assumed to be BLQ in studies without pre-dose concentrations measured. After excluding patients with quantifiable lumefantrine concentrations before the first dose (n = 67/676; 9.9 %), the lowest day 7 dose-adjusted lumefantrine concentrations were measured in capillary blood (Additional file 3: Figure S2).

### Determinants of day 7 lumefantrine concentration

In patients treated with the standard six-dose regimen and without quantifiable lumefantrine concentrations before the first dose (Fig. 1), the independent factors associated with lower day 7 lumefantrine concentrations (Table 2) were: unsupervised (including partially supervised) treatment (43.8 % (95 % CI 38.0 to 49.1 %) lower than supervised treatment); age (infants had 38.1 % (95 % CI 21.7 to 51.1 %), children aged 1–2 years had 41.4 % (95 % CI 32.7 to 48.9 %) and children aged 3–4 years had 20.9 % (95 % CI 9.4 to 30.9 %) lower concentrations than adults); and fever on admission (13.5 % (95 % CI 6.4 to 20.1 %) lower than patients with only a history of fever). As fat was co-administered with artemether-lumefantrine doses in the majority of patients (n = 2,185, 85 %), no fat effect was detected (*P* = 0.943). Within the time window studied (days 6 to 8), the lumefantrine concentration declined by 36.6 % (95 % CI 13.1 to 53.7 %) per day, which corresponds to a terminal half-life of 37 (95 % CI 22 to 118) hours. The effect of body weight-adjusted (mg/kg) dose was relatively small, increasing the day 7 lumefantrine concentration by 9.8 % (95 % CI 4.0 to 15.9 %) for each 20 mg/kg increase in total lumefantrine dose. After adjusting for these factors in a multivariable model (Table 2), assay matrix sampled (venous plasma or capillary or venous blood on filter paper) did not significantly alter the day 7 lumefantrine concentrations.

**Table 2:** Determinants of day 7 lumefantrine concentrations in non-pregnant patients treated with the six-dose artemether-lumefantrine regimen

	N (n)^a^	Change (%) (95 % CI)	*P* value
Univariable model			
Dose (mg/kg)	2,551	0.3 (0.1 to 0.6)	0.010
Co-administration with fat	2,574 (2,185)	−1.0 (−28.2 to 26.2)	0.943
Unsupervised administration^b^	2,574 (816)	−45.4 (−50.6 to −39.6)	<0.001
Age			
< 1 year	82	−37.7 (−50.5 to −21.6)	<0.001
1–2 years	562	−46.5 (−53.5 to −38.5)	<0.001
3–4 years	597	−26.1(−35.7 to −15.1)	<0.001
5–11 years	534	−3.4 (−12.6 to 6.9)	0.507
12+ years	799	Reference	
Sample matrix			
Capillary blood^c^	848	−38.2 (−54.0 to −22.4)	<0.001
Venous blood^c^	544	18.5 (−34.7 to 71.8)	0.458
Venous plasma	1,182	Reference	
Day of sampling^d^	2,574	−36.8 (−56.6 to −17.0)	0.004
Fever^e^	2,426 (1,054)	−17.4 (−24.0 to −10.8)	<0.001
Hemoglobin^f^	2,200	1.0 (−0.8 to 2.8)	0.272
Baseline parasitemia (log_10_)	2,554	−8.8 (−13.3 to −4.1)	<0.001
WAZ^g^	1,240	3.2 (−1.7 to 8.0)	0.191
UWA^h^	1,240 (215)	−4.5 (−19.1 to 10.2)	0.559
Multivariable model^i^			
Dose (mg/kg)	2,422	0.5 (0.2 to 0.7)	0.001
Unsupervised administration^b^	2,422 (795)	−43.8 (−49.1 to −38.0)	<0.001
Day of sampling^d^	2,422	−36.4 (−53.6 to −12.9)	0.005
Fever^e^	2,422 (1,052)	−13.5 (−20.1 to −6.4)	<0.001
Baseline parasitemia (log_10_)	2,422	−5.1 (−9.8 to −0.1)	0.045
Age			
< 1 year	82	−38.1 (−51.1 to −21.7)	<0.001
1–2 years	555	−41.4 (−48.9 to −32.7)	<0.001
3–4 years	594	−20.9 (−30.9 to −9.4)	0.001
5–11 years	508	−6.6 (−16.1 to 4.0)	0.215
12+ years	683	Reference	
Multivariable model – supervised administration only^j^			
Dose (mg/kg)	1,562	0.4 (0.1 to 0.7)	0.007
Day of sampling^d^	1,562	−43.0 (−60.1 to −18.6)	0.001
Sample matrix^k^			
Capillary blood^c^	366	−15.2 (−31.0 to 4.3)	0.119
Venous blood^c^	541	1.4 (−23.2 to 34.0)	0.921
Venous plasma	655	Reference	
Hemoglobin			
Capillary blood^c^	366	−5.4 (−9.0 to −1.7)	0.005
Venous blood^c^	541	2.4 (−1.5 to 6.4)	0.229
Venous plasma	655	−1.8 (−4.2 to 0.7)	0.163
Fever^e^	1,562 (590)	−12.2 (−19.3 to −4.4)	0.003
Baseline parasitemia (log_10_)	1,562	−6.1 (−10.7 to −1.4)	0.012
Age			
< 3 years old UWA^h^	28	−52.8 (−65.0 to −36.5)	<0.001
Not UWA	262	−38.6 (−47.5 to −28.2)	<0.001
3–4 years old UWA^h^	48	−19.5 (−35.8 to 1.0)	0.061
Not UWA	229	−17.5 (−29.4 to −3.6)	0.015
5–11 years old	399	−2.0 (−11.5 to 8.5)	0.881
≥ 12 years old	596	Reference	

^a^N, total number of patients with non-missing data; n, number of patients with this characteristic; ^b^unsupervised administration includes five studies with no supervised doses and two studies with the three morning doses supervised; ^c^collected on filtered paper; ^d^per day, evaluated between days 6 and 8; ^e^defined as axillary temperature >37.5 °C on enrolment; ^f^no statistically significant association was found when stratified by sample matrix; *P* for interaction = 0.435; ^g^World Health Organization (WHO) Child Growth Standards weight-for-age Z-score (WAZ) in children <5 years of age; ^h^defined using a WAZ < −2 in children <5 years of age; ^i^151 out of 2,574 patients were excluded from this model due to missing information on dose (23) and fever (148); ^j^131 out of 1,758 patients with supervised treatment were excluded from this model due to missing information on dose (3), fever (127), UWA status (1) or hemoglobin (65); ^k^comparison at mean value of hemoglobin of 11 g/dL

**Fig. 2:**
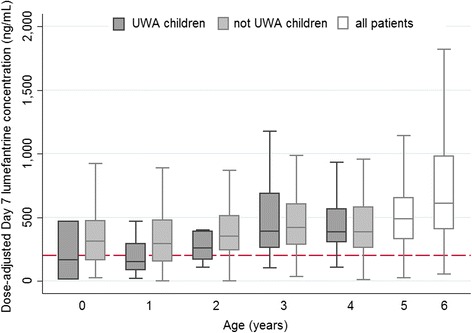
Measured day 7 lumefantrine concentrations in patients given supervised treatment with the recommended six-dose artemether-lumefantrine regimen, by age and nutrition status. Concentrations are dose-adjusted and scaled for a total dose of 72 mg/kg (after excluding patients with quantifiable lumefantrine concentrations pre-treatment). Outside values are not shown

Among patients given supervised treatment with a standard six-dose regimen (Table 2), after adjusting for the other covariates, including body weight-adjusted (mg/kg) dose, adequately nourished children aged 3–4 years had 17.5 % (95 % CI 3.6 to 29.4 %) lower concentrations than adults, while those aged <3 years had 38.6 % (95 % CI 28.2 to 47.5 %) lower concentrations. The effect of nutritional status was apparent in children <3 years of age; those who were underweight had a 23.2 % (95 % CI −0.7 to 41.4 %) lower concentration than adequately nourished children in the same age group (*P* = 0.057) and a 52.8 % (95 % CI 36.5 to 65.0 %) lower concentration than all adults (*P* <0.001). However, this association of lumefantrine concentration with nutritional status was not observed in children aged 3 or 4 years old (*P* = 0.881). Very young children, particularly those who were underweight, not only had lower lumefantrine concentrations compared to older patients for a given total mg/kg dose (Fig. 2, Table 2), but these lower concentrations occurred despite their actual mg/kg dose administered being higher (Additional file 2: Figure S1). Among the 318 children under 3 years of age with supervised treatment administration, 91 (29 %) had day 7 concentrations below 200 ng/ml compared to 129/1,440 (9 %) older children and adults. This risk was highest among the underweight children under 3 years of age, of whom 13/31 (42 %) had day 7 concentrations below 200 ng/ml. The lower concentrations measured in this age group were consistent across regions and assay matrices.

### Day 7 lumefantrine concentration and clinical outcome

After excluding one study on artemether-lumefantrine retreatment of treatment failures and patients without a defined treatment outcome, the association between day 7 lumefantrine concentrations and treatment response was evaluated in 2,528 patients in 20 studies (Fig. 1). Protocol follow-up time varied between 28 and 63 days; 101 patients (3.9 %) were lost to follow-up before day 28. During the follow-up period, 564 recurrent parasitemias were recorded: 321 within 28 days of follow-up; 221 between days 29 and 42; and 62 between days 43 and 63. Among these, there were: 73 *P. falciparum* recrudescences; 376 new *P. falciparum* infections (196 after day 28); 112 *P. vivax* infections; and three infections with another *Plasmodium* species. Forest plots showing individual study estimates of the risks of recrudescence and reinfection by day 28 and day 42 are presented in Additional file 6: Figure S4 and Additional file 7: Figure S5, respectively.

### *P. falciparum* recrudescence

**Fig. 3:**
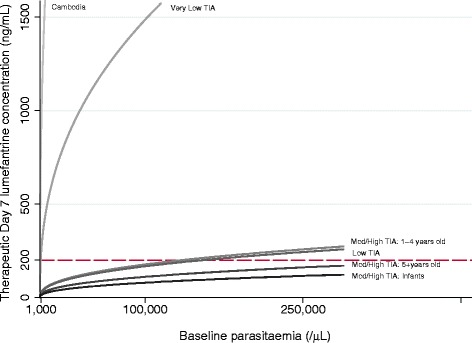
Predicted day 7 lumefantrine concentrations needed to achieve a 95 % cure rate by day 42. Results are derived from multivariable Cox regression model (Table 3) for key patient populations. A zero study site effect was assumed. TIA, transmission intensity area

The main predictors of recrudescence were high baseline parasitemia and low lumefantrine concentration on day 7 (Fig. 3, Table 3). The estimates of the hazard ratios (HRs) for lumefantrine concentration and parasitemia were very robust; the HR coefficient of variation, after exclusion of one study at a time, was 1.9 % and 5.9 %, respectively.

**Table 3:** Cox regression analysis of risk of recrudescence by day 42

Parameter	Patients^a^	Events^a^	HR (95 % CI)	*P* value
Univariable model				
Day 7 lumefantrine concentration (log_2_)				
All^b^	2,528	70	0.64 (0.55 to 0.74)	<0.001
Venous plasma	1,336	46	0.56 (0.46 to 0.69)	<0.001
Capillary blood^b^	663	21	0.68 (0.54 to 0.84)	0.001
Venous blood^b^	529	3	0.84 (0.16 to 4.34)	0.559
Age (years)	2,528	70	1.01 (0.99 to 1.04)	0.22
Age category^b^				
< 1 year	73	2	0.32 (0.06 to 1.59)	0.162
1–4 years	1,004	29	0.55 (0.23 to 1.31)	0.177
5–11 years	540	4	0.39 (0.13 to 1.21)	0.103
12+ years	911	32	Reference	
Sex (female)	2,528 (1,157)	70 (32)	1.33 (0.82 to 2.16)	0.254
Baseline parasitemia (log_10_)	2,527	70	1.85 (1.33 to 2.76)	0.003
Baseline temperature (°C)	2,384	56	1.17 (0.93 to 1.46)	0.181
Baseline fever^c^	2,384 (1,049)	56 (36)	1.49 (0.84 to 2.64)	0.177
Baseline gametocytemia	2,197 (109)	53 (4)	1.69 (0.58 to 4.94)	0.335
Hemoglobin^b^	2,189	57	1.01 (0.90 to 1.14)	0.857
Moderate anemia	2,279 (1,022)	58 (25)	0.78 (0.44 to 1.39)	0.402
Severe anemia	2,279 (138)	58 (6)	1.23 (0.51 to 2.99)	0.642
WAZ	1,076	34	0.92 (0.70 to 1.21)	0.538
Underweight ^d^	1,076	34	1.36 (0.61 to 3.03)	0.457
Palpable spleen	1,732 (272)	35 (8)	1.25 (0.56 to 2.78)	0.581
Palpable liver	1,732 (102)	35 (5)	1.19 (0.43 to 3.29)	0.732
<Six-dose artemether-lumefantrine regimen	2,528 (142)	70 (15)	5.32 (1.97 to 14.39)	<0.001
D2 Parasite positive count	1,901 (179)	47 (17)	3.12 (1.55 to 6.25)	0.001
D3 Parasite positive count	2,153 (23)	51 (5)	5.29 (1.78 to 15.76)	0.003
Log of PRR48^e^	1,897	47	0.86 (0.76 to 0.97)	0.011
Early parasitological response^f^				
Fast	1,193	36	Reference	
Slow	65	8	2.98 (1.25 to 7.07)	0.014
Unknown	1,270	26	1.05 (0.54 to 2.06)	0.879
Transmission intensity areas (TIA)^b^				
Very low	66	9	6.52 (1.28 to 33.23)	0.024
Low	500	23	1.74 (0.59 to 5.09)	0.314
Moderate	738	12	0.83 (0.23 to 2.94)	0.769
High	1,224	26	Reference	
Multivariable model^g^				
Day 7 lumefantrine concentration (log_2_)	2,527	70	0.63 (0.54 to 0.73)	<0.001
Baseline parasitemia (log_10_)	2,527	70	1.87 (1.22 to 2.87)	0.004
Location/transmission intensity area (TIA)				
Cambodia	77	10	13.44 (2.51 to 72.02)	0.002
Very low TIA	66	9	5.63 (1.10 to 28.92)	0.039
Low TIA	423	13	1.32 (0.34 to 5.07)	0.69
High/moderate TIA				
< 1 year	71	2	0.80 (0.14 to 4.73)	0.807
1–4 years	973	31	1.37 (0.46 to 4.11)	0.578
5+ years	917	5	Reference	
<Six-dose artemether-lumefantrine regimen	2,527	70	4.38 (1.54 to 12.49)	0.006
Early parasitological response				
Fast	1,193	36	Reference	
Slow	65	8	2.72 (1.04 to 7.16)	0.042
Unknown	1,269	26	1.02 (0.51 to 2.04)	0.964

^a^Numbers in brackets refer to patients with the symptom/characteristic present; ^b^ *P* value for testing the proportional hazards assumption <0.05; ^c^fever defined as axillary temperature >37.5 °C; ^d^only evaluated in children <5 years of age and defined as weight-for-age Z-score (WAZ) < −2; ^e^PRR48, parasite reduction rate at 48 hours; ^f^slow early parasitological response was defined as parasite positivity on day 3 or log_e_ of PRR48 < 5 (provided positive count was recorded on day 2); ^g^1 out of 2,528 patients with outcome were excluded from this model due to missing information on baseline parasitemia. TIA, transmission intensity area

In the multivariable model (Table 3), the highest risk of recrudescence (HR 13.44; 95 % CI 2.51 to 72.02; *P* = 0.002) was observed in one study in a low transmission setting in Cambodia (2003–2004; n = 79), where delayed early parasitological response was observed and artemisinin resistance subsequently confirmed. An increased risk of recrudescence (HR 5.63; 95 % CI 1.10 to 28.92; *P* = 0.039) was also observed in the very low transmission areas studied (n = 66, two studies, both in Thailand). The increased risk of recrudescence in these areas was still observed after adjusting for slow early parasitological responses in 65 of 2,527 patients, who had an almost 3-fold higher risk of recrudescence (HR 2.72; 95 % CI 1.04 to 7.16; *P* = 0.014) compared to those with faster parasite clearance.

Within the low transmission areas, the small number of recrudescences precluded any meaningful comparisons between age categories. In the moderate/high transmission areas, the increased risk of recrudescence in children aged 1–4 years old was not statistically significant (HR 1.37; 95 % CI 0.46 to 4.11). However, after adjusting for their day 7 lumefantrine concentrations, the risk of recrudescence appeared to increase with a decrease in the weight-for-age Z-score (WAZ), but this did not reach statistical significance (HR 1.26; 95 % CI 0.94 to 1.69; *P* = 0.12 per unit decrease, which corresponds to a doubling of the recrudescence risk (HR 2.01; 95 % CI 0.83 to 4.83) for a young child with a WAZ of −3, when compared to an adequately nourished child with a WAZ of 0).

This multivariable model (Table 3) predicts that a day 7 lumefantrine concentration of at least 200 ng/ml was sufficient to achieve 95 % cure rates in all patients, including infants, in low, moderate and high transmission intensity areas, provided the baseline parasitemia was below 135,000 parasites/μl. However, in Cambodia and the areas of very low transmission intensity studied, concentrations ≥1,000 ng/ml (depending on baseline parasitemia) were required (Fig. 3).

### *P. falciparum* re-infection

A 2-fold increase in (or doubling of) day 7 lumefantrine concentrations was associated with a 30 % reduction in the risk of reinfection (Table 4); however, this was not constant over the follow-up period. The effect of day 7 lumefantrine concentration remained apparent for longer when measured in plasma than if measured in capillary or venous blood collected on filter paper (28 versus 21 days), which is consistent with the lower sensitivity of the filter paper assays (Additional file 1: Table S1). Not surprisingly given lumefantrine’s elimination half-life, no association between day 7 concentration and the risk of reinfection was observed after day 28 (HR 1.02; 95 % CI 0.90 to 1.16; *P* = 0.735). These results were confirmed by the sensitivity analyses, when each study was excluded in turn.

**Table 4:** Cox regression analysis of risk of new *Plasmodium falciparum* infection by day 28

Parameter	Patients^a^	Events^a^	HR (95 % CI)	*P* value
Univariable model				
Day 7 lumefantrine concentration (log_2_)				
All^b^	2,528	180	0.79 (0.72 to 0.87)	<0.001
Venous plasma	1,336	40	0.65 (0.56 to 0.76)	<0.001
Capillary blood^b^	663	130	0.87 (0.78 to 0.98)	0.002
Venous blood	529	10	0.82 (0.33 to 2.07)	0.619
Age (years)	2,528	180	0.95 (0.92 to 0.99)	0.009
Age category				
< 1 year	73	9	3.51 (1.16 to 10.59)	0.026
1–4 years	1,004	148	5.01 (2.06 to 12.21)	<0.001
5–11 years	540	14	2.95 (1.17 to 7.39)	0.021
12+ years	911	9	Reference	
Sex (female)	2,528 (1,157)	180 (93)	1.13 (0.84 to 1.51)	0.428
WAZ	1,076	157	0.86 (0.76 to 0.97)	0.015
Underweight^c^	1,076 (192)	157 (37)	1.22 (0.84 to 1.79)	0.299
Transmission intensity areas (TIAs)				
Very low/low^d^	557	9	0.17 (0.05 to 0.51)	0.002
Moderate	721	17	0.16 (0.05 to 0.56)	0.004
High	1,070	154	Reference	
Multivariable model				
Day 7 lumefantrine concentration (log_2_)^e^	2,528	180	0.70 (0.63 to 0.78)	<0.001
Location/transmission intensity area (TIA)				
Very low/low/moderate TIA	1,304	26	0.22 (0.06 to 0.82)	0.024
High TIA				
< 1 year	57	9	0.91 (0.27 to 3.05)	0.882
1–4 years	703	138	1.35 (0.49 to 3.74)	0.565
5+ years	464	7	Reference	

^a^Number in brackets refers to patients with presence of the symptom/characteristic; ^b^ *P* value for testing proportional hazards assumption <0.05; ^c^only evaluated in children <5 years of age and defined as weight-for-age Z-score (WAZ) < −2; ^d^very low and low TIAs were combined as no new infections were observed in very low TIA; ^e^for concentrations measured on filter paper, the association with risk of new infection diminished after 21 days, hazard ratio = 1.062 (0.887–1.270), while for venous plasma this remained the same over the whole period. TIA, transmission intensity area

**Fig. 4:**
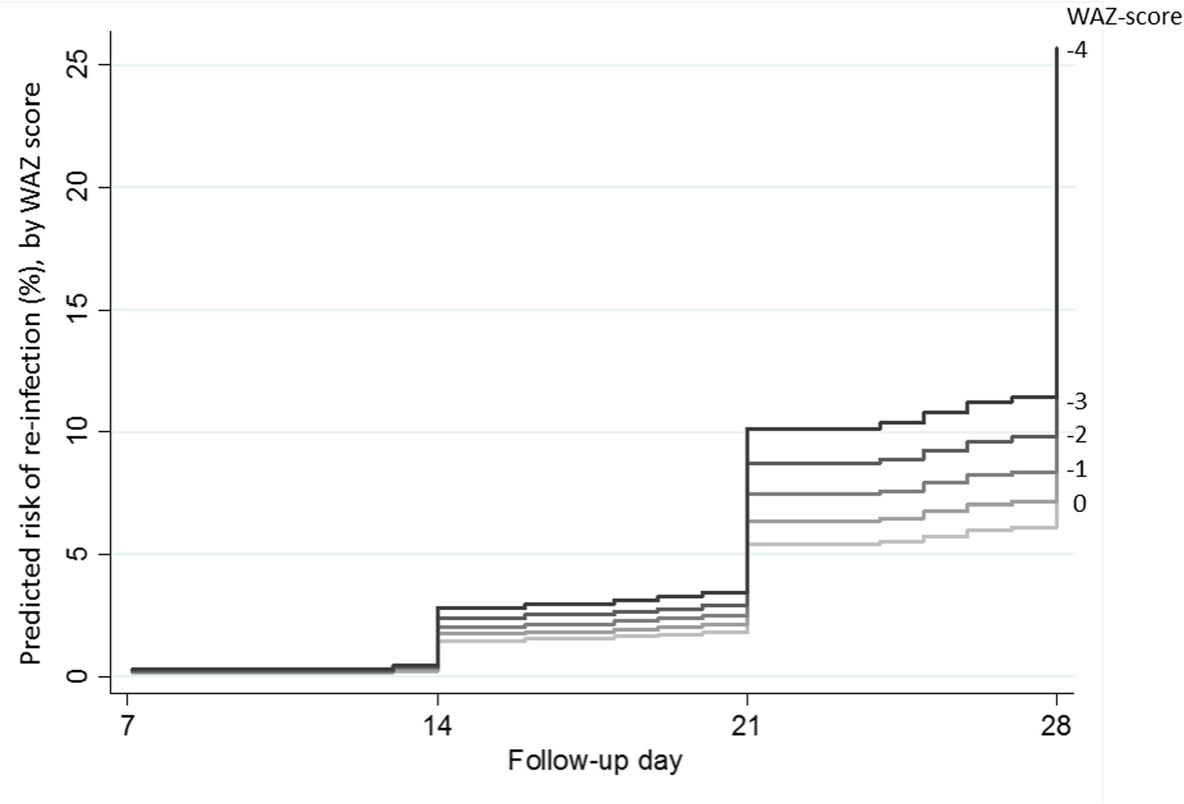
Predicted reinfection rates by day 28 for a day 7 venous plasma lumefantrine concentration of 200 ng/ml. Results are shown for children 1–4 years of age in high transmission intensity areas, depending on their nutrition status (weight-for-age Z-score, WAZ). A zero study site effect was assumed.

As expected, patients in high transmission areas had a higher risk of reinfection overall than patients in other areas (HR 5.76; 95 % CI 2.29 to 14.49; *P* <0.001), after adjusting for the day 7 lumefantrine concentration. Among children 1–4 years of age in high transmission areas, the risk of reinfection increased with a decrease in WAZ (HR 1.18; 95 % CI 1.03 to 1.35 per unit change; *P* = 0.017). This corresponds to a HR of 1.63 (95 % CI 1.09 to 2.44) for a child with WAZ of −3 compared to an adequately nourished child (WAZ = 0). In high transmission intensity areas, the estimated reinfection rates in young children with a day 7 plasma lumefantrine concentration of 200 ng/ml varied from 14 % for children with WAZ of 0 to 17, 19, 22 and 26 % for children with WAZ of −1, −2, −3 and −4, respectively (Fig. 4). In this group of young children, age was not associated with the risk of reinfection (*P* = 0.341).

### Assessment of risk of bias

The risk of bias in individual studies was classified as low (Additional file 8: Table S3). Non-randomized or unblinded studies were included in this analysis as this is not considered a significant risk of bias in PK-PD studies which explore the relationship between drug concentrations and treatment response within a single treatment arm. For these study designs, baseline characteristics across the studies and the process of participant selection, as well as the details of the intervention (drug, dose and duration) are more relevant in considering the potential impact of bias on the results. In terms of assessment of the drug concentrations, the blinding of all three independent outcome laboratory assessments (namely the pharmacokinetic assays that measure the day 7 lumefantrine concentrations, as well as the microscopy and PCR laboratories that classify treatment outcome) further reduce the risk of bias.

Sensitivity analyses showed that exclusion of any of the studies (including those that are non-randomized or unblinded, or used different lumefantrine assay methods) did not change the main conclusions of the analysis (coefficient of variation was 1.9 % and 5.9 % for lumefantrine concentration and parasitemia, respectively). Similarly, the results were not affected by the exclusion of patients with missing or indeterminate PCR data (multiple imputation estimates of HR were 0.65 (95 % CI 0.56 to 0.76) and 1.76 (95 % CI 1.17 to 2.67) for log_2_ concentration and log_10_ parasitemia, respectively).

Five studies (Additional file 9: Table S4) that were not included in the pooled analysis represent 447 patients with day 7 concentrations measured and 14 of the recrudescent infections observed during follow-up. These represent only 14 % and 16 % of the sample numbers in all 31 studies identified, respectively. It is unlikely that inclusion of these would have affected the results significantly, as the aggregate data reported on their baseline characteristics, day 7 lumefantrine concentrations and risks of treatment failure were similar to those included in our meta-analysis.

## Discussion

In this study, the largest pooled analysis of individual patient PK-PD data for any antimalarial to date, artemether-lumefantrine was generally highly effective with only 73 (3 %) *P. falciparum* recrudescences among the 2,528 patients included in the treatment outcome analysis. The most important determinants of therapeutic response were baseline parasite density and day 7 blood or plasma lumefantrine concentrations. Current artemether-lumefantrine dosing recommendations achieve day 7 lumefantrine concentrations ≥200 ng/ml and >98 % cure rates in most uncomplicated malaria patients. However, three groups were at increased risk of treatment failure: very young children, particularly those that are underweight-for-age; patients with high parasitemias; and patients in areas with very low transmission intensity and slow early parasitological responses (reflecting artemisinin resistance).

Young children had 17.5–52.8 % lower day 7 lumefantrine concentrations following supervised treatment despite their actual mg/kg dose being higher, as they have higher body weight normalized apparent clearance after oral administration [35]. Optimal dosing of artemether-lumefantrine in young children requires urgent investigation. Children under 5 years of age are at particular risk as they account for 78 % of all malaria-related deaths [4]. Although this large pharmacokinetic data set did not have sufficient recrudescences to confirm the trend towards a higher risk of recrudescence among underweight young children, this was confirmed in the larger WWARN artemether-lumefantrine dose impact analysis. Underweight African children between 1 and 3 years old had an increased risk of recrudescence when compared with those of the same age who were not underweight (adjusted HR 1.66; 95 % CI 1.05 to 2.63; *P* = 0.028) and a 4-fold higher risk than patients aged ≥12 years (adjusted HR 4.05; 95 % CI 1.78 to 9.18; *P* = 0.001) [67]*.*

Malaria and malnutrition are common co-morbidities, particularly in Sub-Saharan Africa, where 90 % of global malaria deaths occur [4] and 30–33 % of children under 5 years of age are underweight [68]. However, there have been few studies on the effect of malnutrition on malaria, and these have yielded conflicting results [69–71]. The mechanisms underlying the effects of malnutrition on antimalarial treatment response are complex and poorly understood. Malnutrition has also been shown to compromise the efficacy of chloroquine, sulfadoxine-pyrimethamine, amodiaquine, dihydroartemisinin and piperaquine [72–75]. Several physiological changes can occur with malnutrition that may decrease drug concentrations, including reduced drug absorption and/or an increased volume of distribution. Malnutrition may reduce protein binding and increase clearance, but concomitant hepatic dysfunction may reduce the metabolism of some drugs. The net effect is uncertain [1, 76]. In addition, the innate and adaptive immune responses may be impaired by malnutrition and micronutrient deficiencies [70, 77, 78], which could explain the increased risk of malaria recurrence observed in our underweight young children even after adjusting for their total day 7 lumefantrine concentrations (unfortunately unbound lumefantrine concentrations were not measured in any of the studies included). A limitation of this study is that we were unable to use the preferred anthropometric indices for determining nutritional status [79]. As the studies pooled for this analysis were designed to assess antimalarial efficacy, most only recorded body weight on a single occasion and height was only recorded in <5 % of young children. Thus we were unable to differentiate acute under-nutrition (low weight-for-height or BMI-for-age) from chronic under-nutrition (low height-for-age), or distinguish tall, thin children from short, well-proportioned children.

At currently recommended doses, the absorption of lumefantrine appears close to saturation [40], or constrained by limited solubility. This was confirmed by the small effect of body weight-adjusted (mg/kg) dose in our study. Thus a simple increase in the number of tablets given at each twice daily dose may not ensure adequate lumefantrine exposure. Administering the same recommended six doses of artemether-lumefantrine over 5 days, dosing at 0, 8, 24, 48, 72 and 96 hours, has been shown to increase the area under the lumefantrine concentration time-curve (AUC) in Asian adults [6, 7, 30], but this may compromise adherence. Further studies of higher, more frequent, or prolonged dosage regimens are needed to determine which dosing adjustments would ensure that all young children, including those that are underweight, could safely achieve the day 7 concentrations required to achieve ≥95 % cure rates.

Achieving acceptable cure rates is particularly challenging for underweight young children with higher parasite densities (>100,000/uL), who require higher day 7 concentrations (up to 256 ng/ml). Hyperparasitemia is an important source of antimalarial drug resistance [80] and occurs commonly in patients with otherwise uncomplicated malaria. In the large WWARN pooled analysis of 14,327 patients treated with artemether-lumefantrine, 9.5 % had parasite densities above 100,000/uL [67]. This 9.5 % prevalence would be an underestimate of all uncomplicated hyperparasitemia, as the WHO recommends excluding hyperparasitemic patients from therapeutic efficacy studies [59]. To exclude uncomplicated hyperparasitemia, microscopy should be used rather than rapid diagnostic tests when feasible, particularly in very young and underweight children. The administration of at least two doses of parenteral artesunate is the preferred treatment for hyperparasitemic patients [1], and the threshold of >250,000/uL in the current WHO definition of uncomplicated malaria in areas of moderate to high transmission intensity [59] appears too high for very young children, particularly if they are underweight.

The risk of artemether-lumefantrine failure was, as expected, highest in western Cambodia, the epicenter of antimalarial drug resistance [81, 82], where day 7 lumefantrine concentrations >1,616 ng/ml appear necessary to achieve acceptable cure rates even for very low baseline parasite densities (<5,000/μL). It is of concern that patients in the very low transmission areas included in this study also required high lumefantrine concentrations (>1,000 ng/ml) to cure even low parasite densities. In these areas it seems unlikely that artemether-lumefantrine dosage regimens could be adjusted to achieve the predicted lumefantrine exposure needed to ensure acceptable cure rates for parasite densities of up to 100,000/μL (the WHO definition of uncomplicated malaria in areas of low intensity malaria transmission). The very low transmission intensity areas included in this analysis comprised only two small studies in Thailand, and data on the frequency of the *pfmdr1* 86 N allele and copy number in our study were insufficient for determining the extent to which these findings simply reflect high levels of lumefantrine resistance, or whether the lack of immunity in these areas of very low transmission intensity further compromises therapeutic efficacy. The WWARN pooled analysis of the relationship between lumefantrine-resistant polymorphisms in *pfcrt* and *pfmdr1* and artemether-lumefantrine treatment response showed that presence of the *pfmdr1* gene N86 (adjusted HR 4.74; 95 % CI 2.29 to 9.78) and increased *pfmdr1* copy number (adjusted HR 6.52; 95 % CI 2.36 to 17.97) were significant independent risk factors for recrudescence in patients treated with artemether-lumefantrine [83].

Even after adjusting for covariates, including site effects (for Cambodia and the nearby very low intensity transmission areas included in our study) and artemether-lumefantrine (mg/kg) dose, slow early parasitological treatment response more than doubled the risk of recrudescence. Artemether pharmacokinetic data were not available for this pooled analysis, and previous publications have been inconsistent. While some reported that higher artemether or dihydroartemisinin exposure was found to decrease parasite clearance time, others have found no clinically meaningful correlation between exposure and parasite clearance times [44, 84, 85]. Whether due to artemisinin resistance and/or inadequate artemether/dihydroartemisinin exposure, a higher residual parasite biomass remains that the partner lumefantrine is less able to clear. Thus ACT treatment failure rates increase, risking the development and spread of resistance to both the artemisinin and lumefantrine components. The slow parasite clearance rates that characterize artemisinin resistance were originally documented in western Cambodia [81, 82]. Despite containment efforts, artemisinin resistance has now been confirmed in five countries across mainland Southeast Asia [86–88], where a total of 331,551 *P. falciparum* malaria cases were notified in 2013 [4], highlighting the urgent need for novel antimalarials.

The simplicity of collecting a single pharmacokinetic sample per patient as an accurate measure of lumefantrine exposure is very appealing, particularly in remote field study sites with minimal infrastructure. The feasibility of pharmacokinetic sampling is further enhanced by the use of capillary blood specimens dried on filter paper, although this method is more vulnerable to inter-operator variability and the effects of anemia. This pooled analysis shows that this matrix is less optimal, being 2- to 3-fold more variable, and 5-fold less sensitive. However, with the therapeutic threshold of 200 ng/ml, the filter paper limit of quantification of 25 ng/ml should be sufficient for the measurements of day 7, if not later, concentrations. Careful attention to dried blood spot sample collection methods may reduce inter-operator variability.

As the determinants of therapeutic response are multi-factorial, studies of the pharmacokinetics of antimalarial drugs often have inadequate power to define optimal dosage recommendations. Pooled individual patient PK-PD data analysis makes the best use of available data for distinguishing treatment failures resulting from inadequate drug exposure from those caused by drug-resistant parasites. The main limitation of pooling individual patient pharmacokinetic data is differences in assay methods between studies. Only two of the studies included in this pooled analysis [42, 44] used mass spectrometry to determine lumefantrine concentrations; early attempts failed due to matrix effects [40]. More recently, several tandem mass spectrometry methods reported having addressed this issue [42, 51, 89]. The risks of one study compromising the overall results of a pooled analysis decrease as the number of studies included increase; in our sensitivity analysis excluding each study one at a time, no individual study was shown to be influential and the main results were shown to be highly robust. Heterogeneity can be reduced by method standardization following the WHO/WWARN consensus document, *Methods and techniques for assessing exposure to antimalarial drugs in clinical field studies* [90]. The WWARN reference material program and, for more stable antimalarial medicines, external proficiency testing have further contributed to reducing inconsistency between antimalarial assays [91].

Dose optimization is best informed when the pharmacokinetic parameters that drive artemether-lumefantrine exposure, particularly bioavailability (including doses above which absorption becomes saturated), volume of distribution and clearance, are characterized adequately in patients with uncomplicated malaria, including high-risk populations. Thus all available drug concentration-over-time data, and not just day 7 concentrations, need to be analyzed using a population PK-PD model. In collaboration with researchers worldwide, WWARN has obtained data from 4,122 malaria patients with a total of 9,258 lumefantrine concentrations (Fig. 1). This WWARN study group will continue to explore this expanded data set to answer key questions more fully, such as: ‘can Day 7 lumefantrine concentrations serve as a convenient surrogate for AUCs in all key target populations?’; ‘what proportion of treatment failures are explained by inadequate drug exposure?’; and ‘which modified dosage regimens should be investigated for important target populations, such as pregnant women, underweight young children or patients with co-morbid conditions (such as HIV/AIDS), or those who are taking drugs that reduce antimalarial exposure (such as efavirenz or rifampicin)?’.

## Conclusions

This study provides a day 7 blood or plasma concentration threshold for evaluating whether individual treatment failures reflect inadequate drug exposure or parasite resistance, comparing artemether-lumefantrine formulations, and informing optimal artemether-lumefantrine dosing regimens. Although current dosing recommendations are adequate for most patients with acute *P. falciparum* malaria, three patient groups are at increased risk of treatment failure: very young children, particularly those that are underweight-for-age; patients with high parasitemias; and patients in very low transmission intensity areas with emerging parasite resistance. Treatment adherence and response should be monitored more closely, and higher, more frequent, or prolonged dosage regimens need to be evaluated in very young children, particularly if malnourished, and in patients with hyperparasitemia. Novel antimalarials are needed for very low transmission intensity areas with emerging artemisinin resistance.

## Additional files

Additional file 1: Table S1. Summary of included studies of lumefantrine pharmacokinetics in *P. falciparum* malaria patients. (DOCX 28 kb) Additional file 2: Figure S1. Distribution of body weight-adjusted dose (mg/kg) administered by age and nutrition status. (TIFF 81 kb) Additional file 3: Figure S2. Day 7 lumefantrine concentration (ng/ml) by assay matrix. Data shown of non-pregnant patients with uncomplicated *P. falciparum* malaria who received the standard six-dose regimen, adjusted for total mg/kg dose and scaled to a total lumefantrine dose of 72 mg/kg. Outside values are not shown. (TIFF 77 kb) Additional file 4: Table S2. Definition of transmission intensity areas (TIAs). (DOCX 22 kb) Additional file 5: Figure S3. Distribution of day 7 lumefantrine concentrations, adjusted for mg/kg dose. Concentrations shown as measured in (A) venous plasma and (B) dried capillary blood or venous blood spots on filter paper^1^, in individual studies for patients treated with standard six-dose artemether-lumefantrine regimen. Studies are sorted by median lumefantrine concentration. (TIFF 219 kb) Additional file 6: Figure S4. Forest plots of Kaplan–Meier estimates (and 95 % CI) of PCR-confirmed recrudescence rates. Recrudescence rates are estimated by (A) day 28 and (B) day 42. For studies with no recrudescences, the binomial confidence interval was calculated using the Wilson method. Studies are sorted by regimen and the recrudescence rate estimate. Dots denote studies of patients given the WHO recommended six-dose artemether-lumefantrine (AL) regimen and squares represent studies with a non-standard AL regimen; light gray squares are studies with four doses of AL over 2 days and dark gray squares are studies with daily AL doses given over 3 days. (TIFF 229 kb) Additional file 7: Figure S5. Forest plots of Kaplan–Meier estimates (and 95 % CI) of PCR-confirmed reinfection rates. Reinfection rates are estimated by (A) day 28 and (B) day 42. For studies with no reinfections, the binomial confidence interval was calculated using the Wilson method. Studies are sorted by transmission intensity and the reinfection rate estimate. Dots denote studies of patients given the WHO recommended six-dose artemether-lumefantrine (AL) regimen and squares represent studies with a non-standard AL regimen; light gray squares are studies with four doses of AL over 2 days and dark gray squares are studies with three doses of AL over 3 days. (TIFF 197 kb) Additional file 8: Table S3. Assessment of risk of bias within studies included in the pooled analysis. Percentage of missing covariates evaluated in the whole pharmacokinetic study, not just in patients with day 7 concentration measured. (XLSX 17 kb) Additional file 9: Table S4. Studies with lumefantrine day 7 concentration measured, but not included in the pooled analysis. Only data for non-pregnant malaria patients with measured concentration on day 7 are presented. (DOCX 26 kb)

## Abbreviations

ACT: Artemisinin-based combination therapy
AUC: Area under the concentration-time curve
BLQ: Below the limit of quantification
BMI: Body mass index
CI: Confidence interval
HR: Hazard ratio
IQR: Interquartile range
LLOQ: Lower limit of quantification
OxTREC: Oxford Tropical Research Ethics Committee
PCR: Polymerase chain reaction
PK: Pharmacokinetic
PK-PD: Pharmacokinetic-pharmacodynamic
PRR48: Parasite reduction rate at 48 hours
TIA: Transmission intensity area
WAZ: Weight-for-age Z-score
WHO: World Health Organization
WWARN: WorldWide Antimalarial Resistance Network

## References

[1] World Health Organization (WHO) (2015). Guidelines for the treatment of malaria.

[2] White NJ, Stepniewska K, Barnes K, Price RN, Simpson J (2008). Simplified antimalarial therapeutic monitoring: using the day-7 drug level?. Trends Parasitol.

[3] Barnes KI, Lindegardh N, Ogundahunsi O, Olliaro P, Plowe CV, Randrianarivelojosia M (2007). World Antimalarial Resistance Network (WARN) IV: clinical pharmacology. Malar J.

[4] World Health Organization (WHO) (2014). World malaria report 2014.

[5] Mwesigwa J, Parikh S, McGee B, German P, Drysdale T, Kalyango JN (2010). Pharmacokinetics of artemether-lumefantrine and artesunate-amodiaquine in children in Kampala, Uganda. Antimicrob Agents Chemother.

[6] White NJ, van Vugt M, Ezzet F (1999). Clinical pharmacokinetics and pharmacodynamics and pharmacodynamics of artemether-lumefantrine. Clin Pharmacokinet.

[7] Ezzet F, van Vugt M, Nosten F, Looareesuwan S, White NJ (2000). Pharmacokinetics and pharmacodynamics of lumefantrine (benflumetol) in acute falciparum malaria. Antimicrob Agents Chemother.

[8] McGready R, Tan SO, Ashley EA, Pimanpanarak M, Viladpai-Nguen J, Phaiphun L (2008). A randomised controlled trial of artemether-lumefantrine versus artesunate for uncomplicated plasmodium falciparum treatment in pregnancy. PLoS Med.

[9] Checchi F, Piola P, Fogg C, Bajunirwe F, Biraro S, Grandesso F (2006). Supervised versus unsupervised antimalarial treatment with six-dose artemether-lumefantrine: pharmacokinetic and dosage-related findings from a clinical trial in Uganda. Malar J.

[10] Rahman MM, Dondorp AM, Day NP, Lindegardh N, Imwong M, Faiz MA (2008). Adherence and efficacy of supervised versus non-supervised treatment with artemether/lumefantrine for the treatment of uncomplicated Plasmodium falciparum malaria in Bangladesh: a randomised controlled trial. Trans R Soc Trop Med Hyg.

[11] Price RN, Uhlemann AC, van Vugt M, Brockman A, Hutagalung R, Nair S (2006). Molecular and pharmacological determinants of the therapeutic response to artemether-lumefantrine in multidrug-resistant Plasmodium falciparum malaria. Clin Infect Dis.

[12] Denis MB, Tsuyuoka R, Lim P, Lindegardh N, Yi P, Top SN (2006). Efficacy of artemether-lumefantrine for the treatment of uncomplicated falciparum malaria in northwest Cambodia. Trop Med Int Heal.

[13] Mosha D, Guidi M, Mwingira F, Abdulla S, Mercier T, Decosterd LA (2014). Population pharmacokinetics and clinical response for artemether-lumefantrine in pregnant and nonpregnant women with uncomplicated Plasmodium falciparum malaria in Tanzania. Antimicrob Agents Chemother.

[14] Kloprogge F, Piola P, Dhorda M, Muwanga S, Turyakira E, Apinan S (2013). Population pharmacokinetics of lumefantrine in pregnant and nonpregnant women with uncomplicated Plasmodium falciparum malaria in Uganda. CPT Pharmacometrics Syst Pharmacol.

[15] Tarning J, McGready R, Lindegardh N, Ashley EA, Pimanpanarak M, Kamanikom B (2009). Population pharmacokinetics of lumefantrine in pregnant women treated with artemether-lumefantrine for uncomplicated Plasmodium falciparum malaria. Antimicrob Agents Chemother.

[16] McGready R, Stepniewska K, Lindegardh N, Ashley EA, La Y, Singhasivanon P (2006). The pharmacokinetics of artemether and lumefantrine in pregnant women with uncomplicated falciparum malaria. Eur J Clin Pharmacol.

[17] Piola P, Nabasumba C, Turyakira E, Dhorda M, Lindegardh N, Nyehangane D (2010). Efficacy and safety of artemether-lumefantrine compared with quinine in pregnant women with uncomplicated Plasmodium falciparum malaria: an open-label, randomised, non-inferiority trial. Lancet Infect Dis.

[18] Ashley EA, Stepniewska K, Lindegardh N, Annerberg A, Kham A, Brockman A (2007). How much fat is necessary to optimize lumefantrine oral bioavailability?. Trop Med Int Heal.

[19] Achan J, Kakuru A, Ikilezi G, Ruel T, Clark TD, Nsanzabana C (2012). Antiretroviral agents and prevention of malaria in HIV-infected Ugandan children. N Engl J Med.

[20] Byakika-Kibwika P, Lamorde M, Mayito J, Nabukeera L, Namakula R, Mayanja-Kizza H (2012). Significant pharmacokinetic interactions between artemether/lumefantrine and efavirenz or nevirapine in HIV-infected Ugandan adults. J Antimicrob Chemother.

[21] Huang L, Parikh S, Rosenthal PJ, Lizak P, Marzan F, Dorsey G (2012). Concomitant efavirenz reduces pharmacokinetic exposure to the antimalarial drug artemether-lumefantrine in healthy volunteers. J Acquir Immune Defic Syndr.

[22] Lamorde M, Byakika-Kibwika P, Mayito J, Nabukeera L, Ryan M, Hanpithakpong W (2013). Lower artemether, dihydroartemisinin and lumefantrine concentrations during rifampicin-based tuberculosis treatment. AIDS.

[23] Lefevre G, Bindschedler M, Ezzet F, Schaeffer N, Meyer I, Thomsen MS (2000). Pharmacokinetic interaction trial between co-artemether and mefloquine. Eur J Pharm Sci.

[24] WorldWide Antimalarial Resistance Network (WWARN) (2013). The WWARN project terms of submission.

[25] WorldWide Antimalarial Resistance Network (WWARN) (2012). Clinical module: data management and statistical analysis plan. Version 1.2.

[26] WorldWide Antimalarial Resistance Network (WWARN) (2011). Pharmacology module: data management and statistical analysis plan (DMSAP). Version 1.0.

[27] Vugt MV, Wilairatana P, Gemperli B, Gathmann I, Phaipun L, Brockman A (1999). Efficacy of six doses of artemether-lumefantrine (benflumetol) in multidrug-resistant Plasmodium falciparum malaria. Am J Trop Med Hyg.

[28] Schramm B, Valeh P, Baudin E, Mazinda CS, Smith R, Pinoges L (2013). Tolerability and safety of artesunate-amodiaquine and artemether-lumefantrine fixed dose combinations for the treatment of uncomplicated Plasmodium falciparum malaria: two open-label, randomized trials in Nimba County. Liberia Malar J.

[29] Dorsey G, Staedke S, Clark TD, Njama-Meya D, Nzarubara B, Maiteki-Sebuguzi C (2007). Combination therapy for uncomplicated falciparum malaria in Ugandan children: a randomized trial. JAMA.

[30] van Vugt M, Looareesuwan S, Wilairatana P, McGready R, Villegas L, Gathmann I (2000). Artemether-lumefantrine for the treatment of multidrug-resistant falciparum malaria. Trans R Soc Trop Med Hyg.

[31] Ngasala BE, Malmberg M, Carlsson AM, Ferreira PE, Petzold MG, Blessborn D (2011). Effectiveness of artemether-lumefantrine provided by community health workers in under-five children with uncomplicated malaria in rural Tanzania: an open label prospective study. Malar J.

[32] Ngasala BE, Malmberg M, Carlsson AM, Ferreira PE, Petzold MG, Blessborn D (2011). Efficacy and effectiveness of artemether-lumefantrine after initial and repeated treatment in children <5 years of age with acute uncomplicated Plasmodium falciparum malaria in rural Tanzania: a randomized trial. Clin Infect Dis.

[33] Karunajeewa HA, Mueller I, Senn M, Lin E, Law I, Gomorrai PS (2008). A trial of combination antimalarial therapies in children from Papua New Guinea. N Engl J Med.

[34] Ursing J, Kofoed PE, Rodrigues A, Blessborn D, Thoft-Nielsen R, Bjorkman A (2011). Similar efficacy and tolerability of double-dose chloroquine and artemether-lumefantrine for treatment of Plasmodium falciparum infection in Guinea-Bissau: a randomized trial. J Infect Dis.

[35] Salman S, Page-Sharp M, Griffin S, Kose K, Siba PM, Ilett KF (2011). Population pharmacokinetics of artemether, lumefantrine, and their respective metabolites in Papua New Guinean children with uncomplicated malaria. Antimicrob Agents Chemother.

[36] van Vugt M, Brockman A, Gemperli B, Luxemburger C, Gathmann I, Royce C (1998). Randomized comparison of artemether-benflumetol and artesunate-mefloquine in treatment of multidrug-resistant falciparum malaria. Antimicrob Agents Chemother.

[37] Piola P, Fogg C, Bajunirwe F, Biraro S, Grandesso F, Ruzagira E (2005). Supervised versus unsupervised intake of six-dose artemether-lumefantrine for treatment of acute, uncomplicated Plasmodium falciparum malaria in Mbarara, Uganda: a randomised trial. Lancet.

[38] Schramm B, Valeh P, Baudin E, Mazinda CS, Smith R, Pinoges L (2013). Efficacy of artesunate-amodiaquine and artemether-lumefantrine fixed-dose combinations for the treatment of uncomplicated Plasmodium falciparum malaria among children aged six to 59 months in Nimba County, Liberia: an open-label randomized non-inferiority. Malar J.

[39] Borrmann S, Sasi P, Mwai L, Bashraheil M, Abdallah A, Muriithi S (2011). Declining responsiveness of Plasmodium falciparum infections to artemisinin-based combination treatments on the Kenyan coast. PLoS One.

[40] Ashley EA, Stepniewska K, Lindegardh N, McGready R, Annerberg A, Hutagalung R (2007). Pharmacokinetic study of artemether-lumefantrine given once daily for the treatment of uncomplicated multidrug-resistant falciparum malaria. Trop Med Int Health.

[41] Mayxay M, Khanthavong M, Lindegardh N, Keola S, Barends M, Pongvongsa T (2004). Randomized comparison of chloroquine plus sulfadoxine-pyrimethamine versus artesunate plus mefloquine versus artemether-lumefantrine in the treatment of uncomplicated falciparum malaria in the Lao People’s Democratic Republic. Clin Infect Dis.

[42] Hodel EM, Kabanywanyi AM, Malila A, Zanolari B, Mercier T, Beck HP (2009). Residual antimalarials in malaria patients from Tanzania--implications on drug efficacy assessment and spread of parasite resistance. PLoS One.

[43] Faucher JF, Aubouy A, Adeothy A, Cottrell G, Doritchamou J, Gourmel B (2009). Comparison of sulfadoxine-pyrimethamine, unsupervised artemether-lumefantrine, and unsupervised artesunate-amodiaquine fixed-dose formulation for uncomplicated plasmodium falciparum malaria in Benin: a randomized effectiveness noninferiority trial. J Infect Dis.

[44] Djimde AA, Tekete M, Abdulla S, Lyimo J, Bassat Q, Mandomando I (2011). Pharmacokinetic and pharmacodynamic characteristics of a new pediatric formulation of artemether-lumefantrine in African children with uncomplicated Plasmodium falciparum malaria. Antimicrob Agents Chemother.

[45] Lindegardh N, Annerberg A, Blessborn D, Bergqvist Y, Day N, White NJ (2005). Development and validation of a bioanalytical method using automated solid-phase extraction and LC-UV for the simultaneous determination of lumefantrine and its desbutyl metabolite in plasma. J Pharm Biomed Anal.

[46] Zeng MY (1996). Determination of benflumetol in human plasma by reversed-phase high-performance liquid chromatography with ultraviolet detection. J Chromatogr B Biomed Appl.

[47] Blessborn D, Romsing S, Annerberg A, Sundquist D, Bjorkman A, Lindegardh N (2007). Development and validation of an automated solid-phase extraction and liquid chromatographic method for determination of lumefantrine in capillary blood on sampling paper. J Pharm Biomed Anal.

[48] Mansoor SM (1996). Determination of a new antimalarial drug, benflumetol, in blood plasma by high-performance liquid chromatography. J Chromatogr B Biomed Appl.

[49] Hodel EM, Zanolari B, Mercier T, Biollaz J, Keiser J, Olliaro P (2009). A single LC-tandem mass spectrometry method for the simultaneous determination of 14 antimalarial drugs and their metabolites in human plasma. J Chromatogr B Anal Technol Biomed Life Sci.

[50] Wong RP, Salman S, Ilett KF, Siba PM, Mueller I, Davis TM (2011). Desbutyl-lumefantrine is a metabolite of lumefantrine with potent in vitro antimalarial activity that may influence artemether-lumefantrine treatment outcome. Antimicrob Agents Chemother.

[51] Annerberg A, Singtoroj T, Tipmanee P, White NJ, Day NPJ, Lindegardh N (2005). High throughput assay for the determination of lumefantrine in plasma. J Chromatogr B-Analytical Technol Biomed Life Sci.

[52] WorldWide Antimalarial Resistance Network (WWARN) Lumefantrine PK/PD Study Group (2012). Statistical analysis plan. Version 1.0.

[53] Beal SL (2001). Ways to fit a PK model with some data below the quantification limit. J Pharmacokinet Pharmacodyn.

[54] World Health Organization (WHO) (2011). Haemoglobin concentrations for the diagnosis of anaemia and assessment of severity. Vitamin and mineral nutrition information system.

[55] Lee SJ, Stepniewska K, Anstey N, Ashley E, Barnes K, Binh TQ (2008). The relationship between the haemoglobin concentration and the haematocrit in Plasmodium falciparum malaria. Malar J.

[56] WHO Multicentre Growth Reference Study Group (2006). WHO Child Growth Standards: length/height-for-age, weight-for-age, weight-for-length, weight-for-height and body mass index-for-age: methods and development.

[57] Gething PW, Patil AP, Smith DL, Guerra CA, Elyazar IR, Johnston GL (2011). A new world malaria map: Plasmodium falciparum endemicity in 2010. Malar J.

[58] Stepniewska K, Ashley E, Lee SJ, Anstey N, Barnes KI, Binh TQ (2010). In vivo parasitological measures of artemisinin susceptibility. J Infect Dis.

[59] World Health Organization (WHO) (2009). Methods for surveillance of antimalarial drug efficacy.

[60] Glidden DV, Vittinghoff E (2004). Modelling clustered survival data from multicentre clinical trials. Stat Med.

[61] Schoenfeld D (1982). Partial residuals for the proportional hazards regression model. Biometrika.

[62] Royston P, Altman DG (1994). Regression using fractional polynomials of continuous covariate: Parsimonios parametric modelling. Appl Stat.

[63] Little RJA, Rubin D (1987). Statistical analysis with missing data.

[64] Stohrer JM, Dittrich S, Thongpaseuth V, Vanisaveth V, Phetsouvanh R, Phompida S (2004). Therapeutic efficacy of artemether-lumefantrine and artesunate-mefloquine for treatment of uncomplicated Plasmodium falciparum malaria in Luang Namtha Province, Lao People’s Democratic Republic. Trop Med Int Heal.

[65] Na-Bangchang K, Karbwang J, Tasanor U, Thanavibul A, Farkad E, Mull R (1999). Pharmacokinetics of benflumetol given as a fixed combination artemether-benflumetol (CGP 56697) in Thai patients with uncomplicated falciparum malaria. Int J Clin Pharmacol Res.

[66] Hatz C, Soto J, Nothdurft HD, Zoller T, Weitzel T, Loutan L (2008). Treatment of acute uncomplicated falciparum malaria with artemether-lumefantrine in nonimmune populations: a safety, efficacy, and pharmacokinetic study. Am J Trop Med Hyg.

[67] Worldwide Antimalarial Resistance Network (WWARN) AL Dose Impact Study Group (2015). The effect of dose on the antimalarial efficacy of artemether-lumefantrine: a systematic review and pooled analysis of individual patient data. Lancet Infect Dis.

[68] Wojcicki JM (2014). The double burden household in sub-Saharan Africa: maternal overweight and obesity and childhood undernutrition from the year 2000: results from World Health Organization Data (WHO) and Demographic Health Surveys (DHS). BMC Public Health.

[69] Fillol F, Cournil A, Boulanger D, Cisse B, Sokhna C, Targett G (2009). Influence of wasting and stunting at the onset of the rainy season on subsequent malaria morbidity among rural preschool children in Senegal. Am J Trop Med Hyg.

[70] Friedman JF, Kwena AM, Mirel LB, Kariuki SK, Terlouw DJ, Phillips-Howard PA (2005). Malaria and nutritional status among pre-school children: results from cross-sectional surveys in western Kenya. Am J Trop Med Hyg.

[71] Genton B, Al-Yaman F, Ginny M, Taraika J, Alpers MP (1998). Relation of anthropometry to malaria morbidity and immunity in Papua New Guinean children. Am J Clin Nutr.

[72] Olanrewaju WI, Johnson AW (2001). Chloroquine-resistant Plasmodium falciparum malaria in Ilorin, Nigeria: prevalence and risk factors for treatment failure. Afr J Med Med Sci.

[73] Sapak P, Garner P, Baea M, Narara A, Heywood P, Alpers M (1991). Ineffectiveness of amodiaquine against Plasmodium falciparum malaria in symptomatic young children living in an endemic malarious area of Papua New Guinea. J Trop Pediatr.

[74] Verret WJ, Arinaitwe E, Wanzira H, Bigira V, Kakuru A, Kamya M (2011). Effect of nutritional status on response to treatment with artemisinin-based combination therapy in young Ugandan children with malaria. Antimicrob Agents Chemother.

[75] Wolday D, Kibreab T, Bukenya D, Hodes R (1995). Sensitivity of Plasmodium falciparum in vivo to chloroquine and pyrimethamine-sulfadoxine in Rwandan patients in a refugee camp in Zaire. Trans R Soc Trop Med Hyg.

[76] Oshikoya KA, Senbanjo IO (2009). Pathophysiological changes that affect drug disposition in protein-energy malnourished children. Nutr Metab.

[77] Gera T, Sachdev HP (2002). Effect of iron supplementation on incidence of infectious illness in children: systematic review. Br Med J.

[78] Nussenblatt V, Semba RD (2002). Micronutrient malnutrition and the pathogenesis of malarial anemia. Acta Trop.

[79] Gorstein J, Sullivan K, Yip R, de Onis M, Trowbridge F, Fajans P (1994). Issues in the assessment of nutritional status using anthropometry. Bull World Heal Organ.

[80] White NJ, Pongtavornpinyo W, Maude RJ, Saralamba S, Aguas R, Stepniewska K (2009). Hyperparasitaemia and low dosing are an important source of anti-malarial drug resistance. Malar J.

[81] Noedl H, Se Y, Schaecher K, Smith BL, Socheat D, Fukuda MM (2008). Evidence of artemisinin-resistant malaria in western Cambodia. N Engl J Med.

[82] Dondorp AM, Nosten F, Yi P, Das D, Phyo AP, Tarning J (2009). Artemisinin resistance in Plasmodium falciparum malaria. N Engl J Med.

[83] Venkatesan M, Gadalla NB, Stepniewska K, Dahal P, Nsanzabana C, Moriera C (2014). Polymorphisms in Plasmodium falciparum chloroquine resistance transporter and multidrug resistance 1 genes: parasite risk factors that affect treatment outcomes for P. falciparum malaria after artemether-lumefantrine and artesunate-amodiaquine. Am J Trop Med Hyg.

[84] Ezzet F, Karbwang J (1998). Population pharmacokinetics and therapeutic response of CGP 56697 (artemether + benflumetol) in malaria patients. Br J Clin Pharmacol.

[85] Hietala SF, Martensson A, Ngasala B, Dahlstrom S, Lindegardh N, Annerberg A (2010). Population pharmacokinetics and pharmacodynamics of artemether and lumefantrine during combination treatment in children with uncomplicated falciparum malaria in Tanzania. Antimicrob Agents Chemother.

[86] Carrara VI, Lwin KM, Phyo AP, Ashley E, Wiladphaingern J, Sriprawat K (2013). Malaria burden and artemisinin resistance in the mobile and migrant population on the Thai-Myanmar border, 1999–2011: an observational study. PLoS Med.

[87] Phyo AP, Nkhoma S, Stepniewska K, Ashley EA, Nair S, McGready R (2012). Emergence of artemisinin-resistant malaria on the western border of Thailand: a longitudinal study. Lancet.

[88] Ashley EA, Dhorda M, Fairhurst RM, Amaratunga C, Lim P, Suon S (2014). Spread of artemisinin resistance in Plasmodium falciparum malaria. N Engl J Med.

[89] Huang L, Li X, Marzan F, Lizak PS, Aweeka FT (2012). Determination of lumefantrine in small-volume human plasma by LC–MS/MS: using a deuterated lumefantrine to overcome matrix effect and ionization saturation. Bioanalysis.

[90] World Health Organization (WHO)/WorldWide Antimalarial Resistance Network WWARN (2011). Methods and techniques for assessing exposure to antimalarial drugs in clinical field studies. 2010.

[91] Lourens C, Lindegardh N, Barnes KI, Guerin PJ, Sibley CH, White NJ (2014). Benefits of a pharmacology antimalarial reference standard and proficiency testing program provided by the WorldWide Antimalarial Resistance Network (WWARN). Antimicrob Agents Chemother.

