# Erratum to: Artemether-lumefantrine treatment of uncomplicated *Plasmodium falciparum* malaria: a systematic review and meta-analysis of day 7 lumefantrine concentrations and therapeutic response using individual patient data

**DOI:** 10.1186/s12916-016-0757-5

**Published:** 2016-12-20

**Authors:** WorldWide Antimalarial Resistance Network (WWARN) Lumefantrine PK/PD Study Group

**Affiliations:** 1 WorldWide Antimalarial Resistance Network (WWARN), Oxford, UK; 2 Division of Clinical Pharmacology, Department of Medicine, University of Cape Town, Cape Town, South Africa

## Erratum

After publication of the original article [1], it came to the authors’ attention that there is an error in the originally-published version of Fig. 2. The labelling of the x-axis for Fig. 2 is not correct. The last two values of the x-axis should read: “5-11” instead of “5”, and “12+” instead of “6”.

**Fig. 2:**
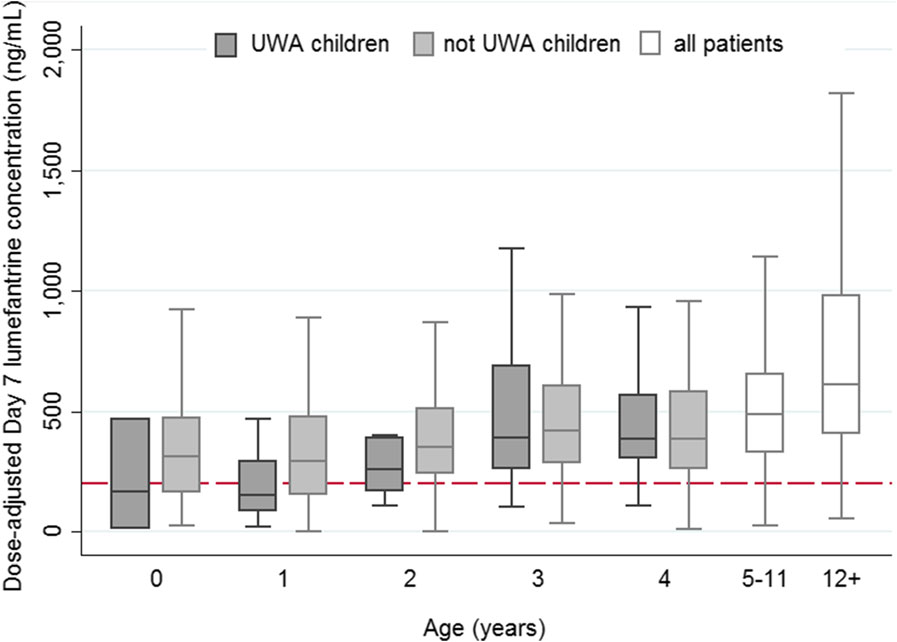
Measured day 7 lumefantrine concentrations in patients given supervised treatment with the recommended six-dose artemether-lumefantrine regimen, by age and nutrition status. Concentrations are dose-adjusted and scaled for a total dose of 72 mg/kg (after excluding patients with quantifiable lumefantrine concentrations pre-treatment). Outside values are not shown

The correct version of Fig. 2 is published in this erratum.
