# Supplementary material for: Artemether-lumefantrine treatment of uncomplicated Plasmodium falciparum malaria: a systematic review and meta-analysis of day 7 lumefantrine concentrations and therapeutic response using individual patient data
Source: BMC Med. 2015 Sep 18;13:227. doi: 10.1186/s12916-015-0456-7 (PMC4574542; doi:10.1186/s12916-015-0456-7)
Supplement: Additional file 1: Table S1. — Summary of included studies of lumefantrine pharmacokinetics in P. falciparum malaria patients. (DOCX 28 kb) [file 12916_2015_456_MOESM1_ESM.docx]

**Table S1.** Summary of included studies of Lumefantrine pharmacokinetics in *Plasmodium falciparum* malaria patients.

|  | | **Study Description** | | | | **Assay Methodology** | | | |
| --- | --- | --- | --- | --- | --- | --- | --- | --- | --- |
| **Country** | **Study** | **Site** | **Year** | **N1/N2*** | **Age** | **Method** | **Matrix** | **LLOQ**  **(ng/ml)** | **Laboratory** |
| **6 doses over 3 days** | | | | | | | | | |
| Benin | EDPJN [44] | Benin | 2006-2007 | 10/110 | 4  (1-7) | LC-MS/MS | venous plasma | 50 | Bioanalytics and Pharmacokinetics department, Novartis Pharma, Rueil Malmaison, France |
|  | YYDSM [43] | Allada, Sekou | 2007 | 44/96 | 2  (1-5) | HPLC-UV [47] | capillary blood on filter paper | 20 | Saint-Louis Hospital biochemistry laboratory, Paris, France. |
| Cambodia | REQES [12] | Battambang | 2003-2004 | 79/79 | 23  (6-49) | HPLC-UV 45] | venous plasma | 24 | Clinical Pharmacology Laboratory, Mahidol-Oxford Tropical Medicine Research Unit, Thailand |
| Guinea Bissau | SXGQP [34] | Bandim, Belem, Cutum | 2007-2008 | 122/191 | 7  (0-15) | HPLC-UV [47] | capillary blood on filter paper | 52.9 | Bioanalytics and Pharmacokinetic laboratory, Dalarna University, Sweden |
| Kenya | EDPJN [44] | Kenya | 2006-2007 | 12/192 | 2  (1-6) | LC-MS/MS | venous plasma | 50 | Bioanalytics and Pharmacokinetics department, Novartis Pharma, Rueil Malmaison, France |
|  | QZJGM [39] | Battambang | 2005 | 101/241 | 3  (1-5) | HPLC-UV [51] | venous plasma | 1.5 | Clinical Pharmacology Laboratory, Mahidol-Oxford Tropical Medicine Research Unit, Thailand |
| Laos | HKNHR [41] | Phalanxay District | 2002 | 77/110 | 12  (1-57) | HPLC-UV [51] | venous plasma | 24 | Clinical Pharmacology Laboratory, Mahidol-Oxford Tropical Medicine Research Unit, Thailand |
| Liberia | FEDZY [28] | Nimba County | 2008-2009 | 438/502 | 13  (6-87) | HPLC-UV [47,49] | venous blood on filter paper | 200 | Service de Pharmacologie Clinique, Hospital St. Vincent de Paul, Paris, France |
|  | UBTXH [38] | Nimba County | 2008-2009 | 106/150 | 3  (1-5) | HPLC-UV [47,49] | venous blood on filter paper | 200 | Service de Pharmacologie Clinique, Hopital St. Vincent de Paul, Paris, France |
| Mali | EDPJN [44] | Mali | 2006 | 8/225 | 3  (2-12) | LC-MS/MS | venous plasma | 50 | Bioanalytics and Pharmacokinetics department, Novartis Pharma, Rueil Malmaison, France |
| Mozambique | EDPJN [44] | Mozambique | 2006 | 11/102 | 3  (1-11) | LC-MS/MS | venous plasma | 50 | Bioanalytics and Pharmacokinetics department, Novartis Pharma, Rueil Malmaison, France |
| Papua New Guinea | RAJDQ [35] | Madang | 2007 | 11/13 | 8  (5-10) | HPLC – UV [50] | venous plasma | 5 | School of Medicine and Pharmacology, University of Western Australia, Australia |
|  | UANQM [33] | Madang, East Sepik | 2005 | 95/128 | 3  (1-5) | HPLC-UV [48] | venous plasma | 5 | School of Medicine and Pharmacology, University of Western Australia, Australia |
| Tanzania | EDPJN [44] | Tanzania | 2006-2007 | 19/269 | 3  (0-7) | LC-MS/MS | venous plasma | 50 | Bioanalytics and Pharmacokinetics department, Novartis Pharma, Rueil Malmaison, France |
|  | GZQDA [32] | Fukayosi, Yombo | 2007-2008 | 353/359 | 3  (0-5) | HPLC-UV [47] | capillary blood on filter paper | 25 | Bioanalytics and Pharmacokinetic laboratory, Dalarna University, Sweden |
|  | KGHRT [32] | Fukayosi, Yombo | 2007-2008 | 152/168 | 3  (0-5) | HPLC-UV [47] | capillary blood on filter paper | 25 | Bioanalytics and Pharmacokinetic laboratory, Dalarna University, Sweden |
|  | UHUBT [42] | Kilombero District | 2008 | 128 [3]/143 | 10  (1-78) | LC-MS/MS [49] | venous plasma | 3 | Division of Clinical Pharmacology, Department de Medicine, University Hospital and University of Lausanne, Lausanne, Switzerland |
|  | XXFCZ [31] | Fukayosi, Yombo | 2007 | 177/244 | 3  (0-5) | HPLC-UV [47] | capillary blood on filter paper | 35 | Bioanalytics and Pharmacokinetic laboratory, Dalarna University, Sweden |
| Thailand | KGJRP [27] | SMRU | 1997-1998 | 66/86 | 20  (3-62) | HPLC-UV [46] | venous plasma | 40 | Novartis Pharma, Basel, Switzerland |
|  | RGPFA [7] | Bangkok | 1996-1997 | 18/32 | 30  (19-40) | HPLC-UV [46] | venous plasma | 40 | Novartis Pharma, Basel, Switzerland |
|  | SAUSX [11,30] | SMRU, Bangkok | 1997 | 134/147 | 22  (2-63) | HPLC-UV [46] | venous plasma | 40 | Novartis Pharma, Basel, Switzerland |
|  | USGDC [40] | SMRU | 2002 | 16/16 | 30  (18-64) | HPLC-UV [51] | venous plasma | 25 | Clinical Pharmacology Laboratory, Mahidol-Oxford Tropical Medicine Research Unit, Thailand |
| Uganda | CCEPC [37] | Mbarara | 2002-2004 | 448 [1]/957 | 9  (1-60) | HPLC-UV [48] | venous plasma | 5 | Bioanalytics and Pharmacokinetics department, Novartis Pharma, Rueil Malmaison, France |
|  | DBCXT [5,29] | Kampala | 2007-2008 | 20/579 | 9  (5-12) | HPLC-UV [51] | venous plasma | 25 | Clinical Pharmacology Laboratory, Mahidol-Oxford Tropical Medicine Research Unit, Thailand |
| **3 doses over 3 days** | | | | | | | | | |
| Thailand | USGDC [40] | SMRU | 2002 | 19/19 | 29  (18-50) | HPLC-UV [51] | venous plasma | 25 | Clinical Pharmacology Laboratory, Mahidol-Oxford Tropical Medicine Research Unit, Thailand |
| **4 doses over 2 days** | | | | | | | | | |
| Thailand | FMNNB [36] | SMRU | 1996 | 43/302 | 25  (9-63) | HPLC–UV [7] | venous plasma | 40 | Novartis Pharma, Basel, Switzerland |
|  | KGJRP [27] | Bangkok | 1996-1997 | 69/85 | 25  (3-62) | HPLC–UV [46] | venous plasma | 40 | Novartis Pharma, Basel, Switzerland |
|  | RGPFA [7] | Bangkok | 1996-1997 | 15/33 | 23  (18-50) | HPLC-UV [46] | Venous plasma | 40 | Novartis Pharma, Basel, Switzerland |

LLOQ: Lower Limit of Quantification; SMRU: Shoklo Malaria Research Unit, LC-MS/MS: liquid chromatography coupled with tandem mass spectrometry; HPLC-UV: High-pressure liquid chromatography with ultraviolet visible detection.

N1/N2 =number of patients in PK study; N2= number of all patients in the study; number in brackets shows number of pregnant women
