# Supplementary figures and images for: Artemether-lumefantrine treatment of uncomplicated Plasmodium falciparum malaria: a systematic review and meta-analysis of day 7 lumefantrine concentrations and therapeutic response using individual patient data
Source: BMC Med. 2015 Sep 18;13:227. doi: 10.1186/s12916-015-0456-7 (PMC4574542; doi:10.1186/s12916-015-0456-7)

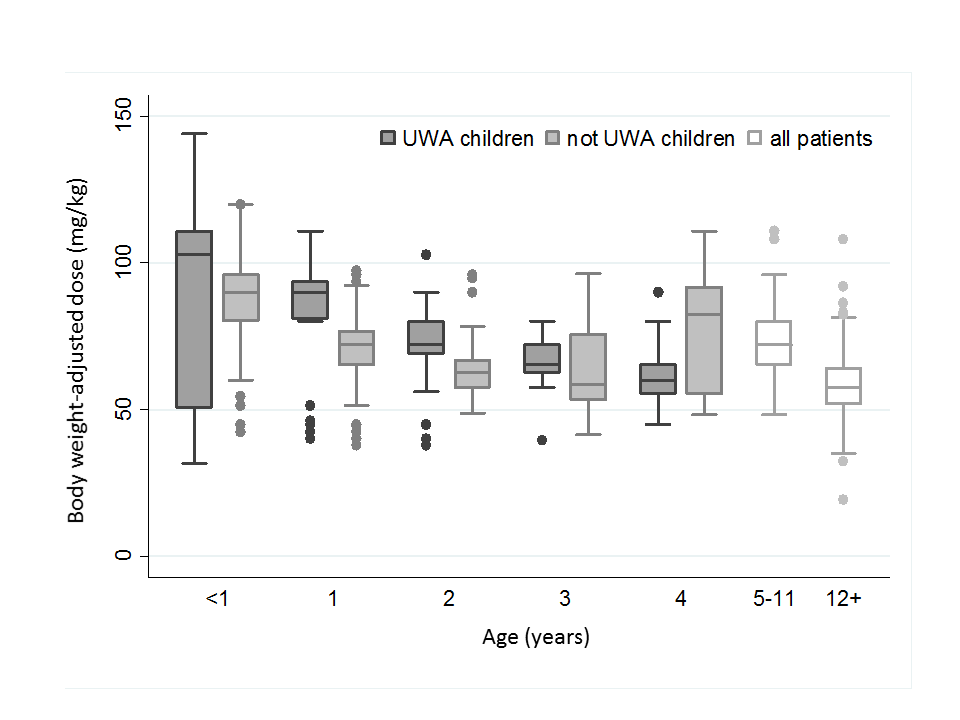

Supplement: Additional file 2: Figure S1. — Distribution of body weight-adjusted dose (mg/kg) administered by age and nutrition status. (TIFF 81 kb) [file 12916_2015_456_MOESM2_ESM.tiff]

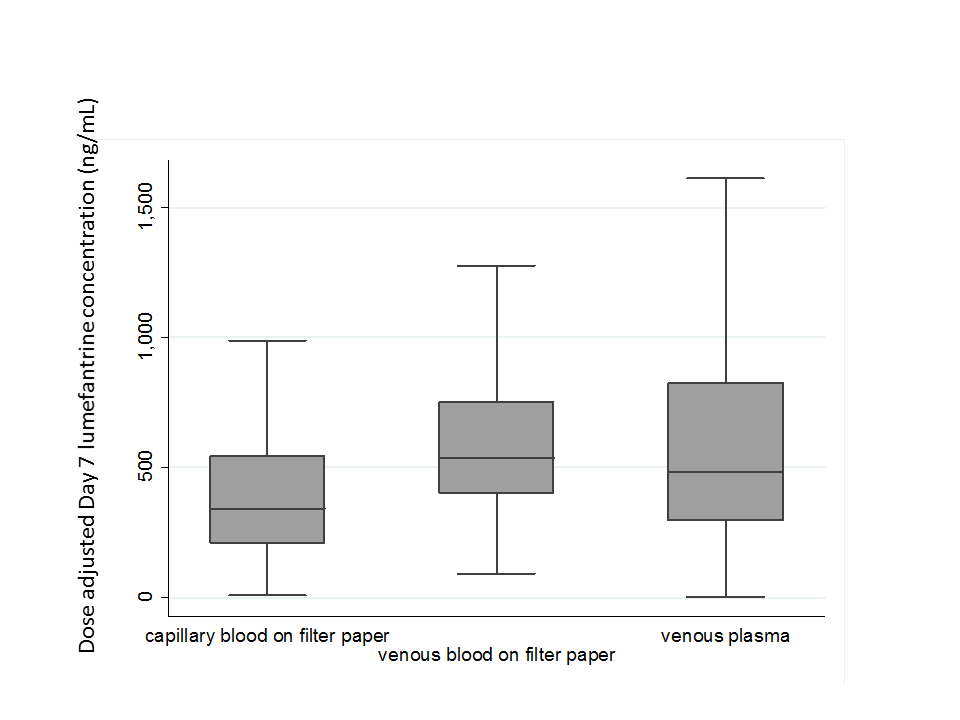

Supplement: Additional file 3: Figure S2. — Day 7 lumefantrine concentration (ng/ml) by assay matrix. Data shown of non-pregnant patients with uncomplicated P. falciparum malaria who received the standard six-dose regimen, adjusted for total mg/kg dose and scaled to a total lumefantrine dose of 72 mg/kg. Outside values are not shown. (TIFF 77 kb) [file 12916_2015_456_MOESM3_ESM.tiff]

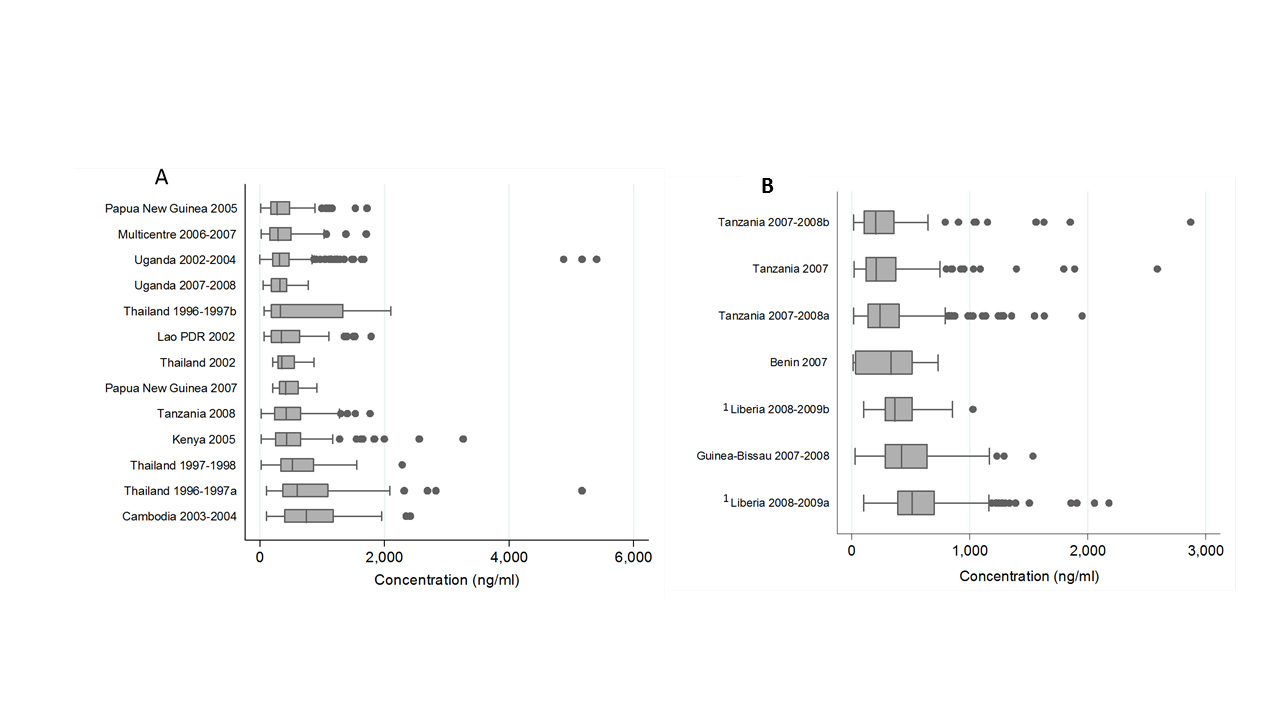

Supplement: Additional file 5: Figure S3. — Distribution of day 7 lumefantrine concentrations, adjusted for mg/kg dose. Concentrations shown as measured in (A) venous plasma and (B) dried capillary blood or venous blood spots on filter paper1, in individual studies for patients treated with standard six-dose artemether-lumefantrine regimen. Studies are sorted by median lumefantrine concentration. (TIFF 219 kb) [file 12916_2015_456_MOESM5_ESM.tiff]

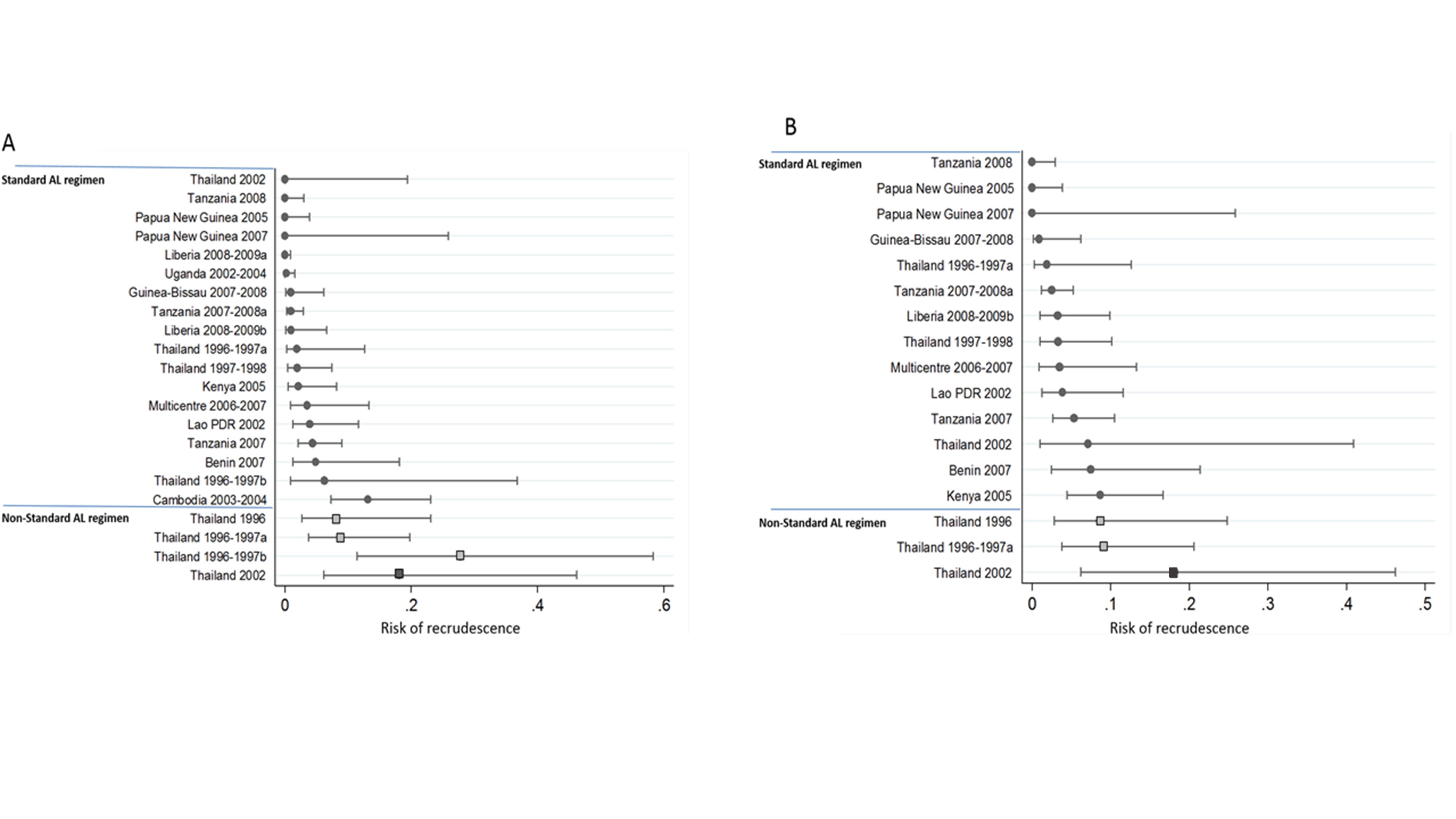

Supplement: Additional file 6: Figure S4. — Forest plots of Kaplan–Meier estimates (and 95 % CI) of PCR-confirmed recrudescence rates. Recrudescence rates are estimated by (A) day 28 and (B) day 42. For studies with no recrudescences, the binomial confidence interval was calculated using the Wilson method. Studies are sorted by regimen and the recrudescence rate estimate. Dots denote studies of patients given the WHO recommended six-dose artemether-lumefantrine (AL) regimen and squares represent studies with a non-standard AL regimen; light gray squares are studies with four doses of AL over 2 days and dark gray squares are studies with daily AL doses given over 3 days. (TIFF 229 kb) [file 12916_2015_456_MOESM6_ESM.jpg]

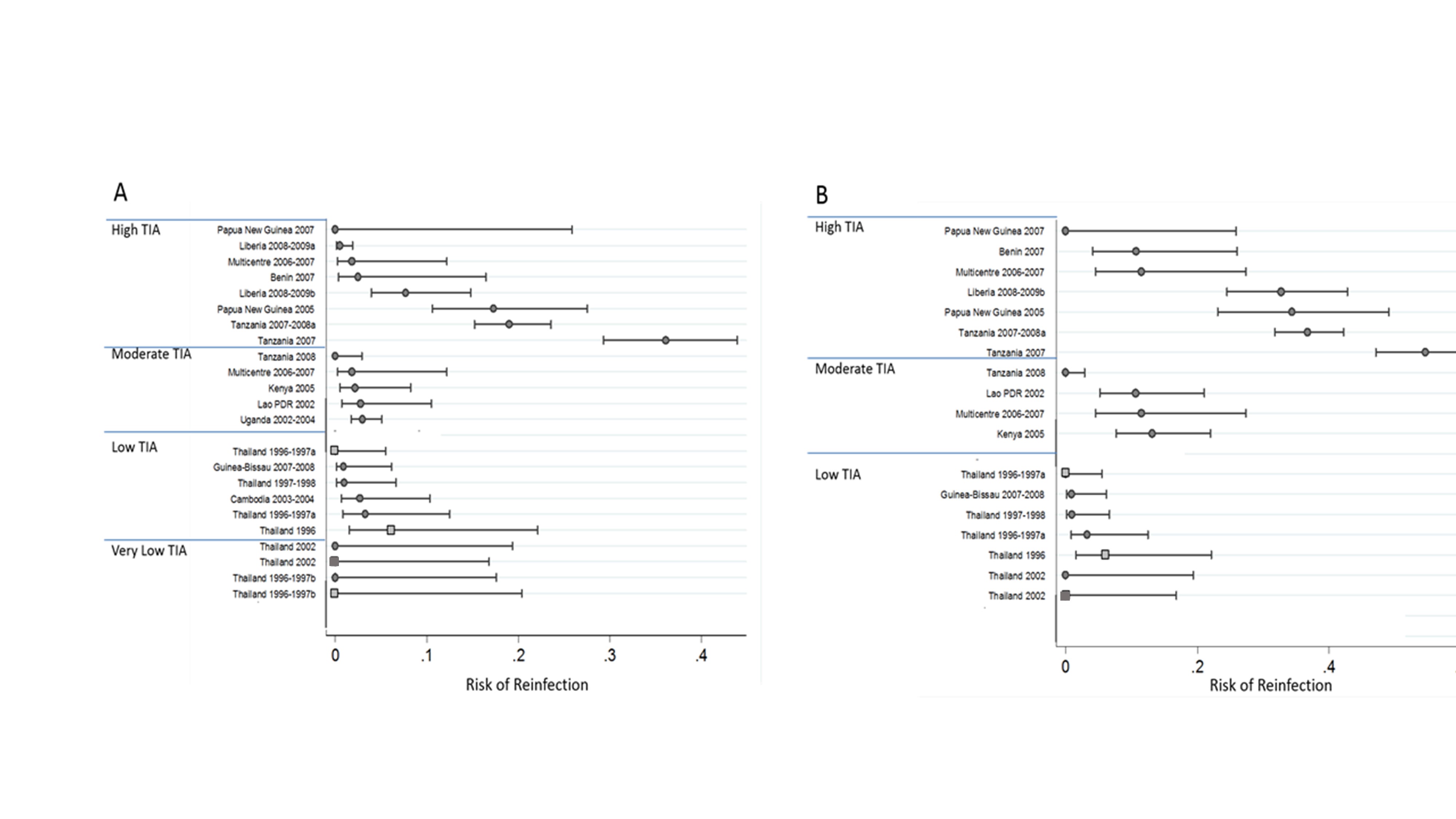

Supplement: Additional file 7: Figure S5. — Forest plots of Kaplan–Meier estimates (and 95 % CI) of PCR-confirmed reinfection rates. Reinfection rates are estimated by (A) day 28 and (B) day 42. For studies with no reinfections, the binomial confidence interval was calculated using the Wilson method. Studies are sorted by transmission intensity and the reinfection rate estimate. Dots denote studies of patients given the WHO recommended six-dose artemether-lumefantrine (AL) regimen and squares represent studies with a non-standard AL regimen; light gray squares are studies with four doses of AL over 2 days and dark gray squares are studies with three doses of AL over 3 days. (TIFF 197 kb) [file 12916_2015_456_MOESM7_ESM.jpg]
