# Supplementary material for: Artemether-lumefantrine treatment of uncomplicated Plasmodium falciparum malaria: a systematic review and meta-analysis of day 7 lumefantrine concentrations and therapeutic response using individual patient data
Source: BMC Med. 2015 Sep 18;13:227. doi: 10.1186/s12916-015-0456-7 (PMC4574542; doi:10.1186/s12916-015-0456-7)
Supplement: Additional file 4: Table S2. — Definition of transmission intensity areas (TIAs). (DOCX 22 kb) [file 12916_2015_456_MOESM4_ESM.docx]

**Table S2.** Definition of Transmission Intensity Areas (TIA).

| **Study Description** | | | | **MAP estimates^1^** | **Study Reinfection Rate(%)^2^**  **(95%CI)** | | **TIA^3^** |
| --- | --- | --- | --- | --- | --- | --- | --- |
| **Country** | **Study**  [Ref] | **Site** | **Year** | **Median**  **(50% Range)** | **Day 28** | **Day 42** |  |
| Benin | YYDSM  [43] | Sekou | 2007 | 0.47  (0.23-0.70) | 3.4  (0.5-22.1) | 14.7  (5.8-34.6) | High |
| Benin | YYDSM  [43] | Allada | 2007 | 0.40  (0.17-0.63) | 9.1  (1.3-49.2) | 9.1  (1.3-49.2) | High |
| Benin | EDPJN  [44] | Benin | 2006  2007 | 0.37  (0.17-0.60)  0.27  (0.10-0.50) | 3.9 (1.5-10.1) | 13.9 (7.9-23.8) | High |
| Cambodia | REQES  [12] | Battambang | 2003 | 0.00  (0.00-0.03) | 2.7 (0.7-10.3) | No data | Low |
| Guinea Bissau | SXGQP  [34] | Bissau | 2006  2007  2008 | 0.07  (0.00-0.17)  0.07  (0.00-0.13)  0.03  (0.00-0.10) | 2.4  (0.9-6.3) | 2.4  (0.9-6.3) | Low |
| Kenya | QZJGM  [39] | Pingilikani | 2005 | 0.47  (0.27-0.63) | 5.4  (3.1-9.2) | 15.0  (10.9-20.4) | Moderate |
| Kenya | EDPJN  [44] | Kenya | 2006  2007 | 0.30  (0.17-0.43)  0.07  (0.03-0.13) | 14.1  (9.7-20.3) | 28.6  (22.4-36.1) | High |
| Lao PDR | HKNHR  [41] | Phalanxay | 2002 | 0.00  (0.00-0.03) | 5.8  (2.6-12.4) | 12.0 (7.0-20.2) | Moderate |
| Liberia | FEDZY  [28] | Liberia | 2008  2009 | 0.37  (0.17-0.60)  0.33  (0.20-0.53) | 1.6  (0.8-3.3) | No data | High |
| Liberia | UBTXH  [38] | Liberia | 2008  2009 | 0.53  (0.30-0.70)  0.53  (0.30-0.70) | 10.4  (6.4-16.7) | 36.8  (29.4-45.5) | High |
| Mali | EDPJN  [44] | Mali | 2006  2007 | 0.20  (0.10-0.37)  0.23  (0.10-0.43) | 13.7  (9.8-19.2) | 26.5  (20.8-33.4) | High |
| Mozambique | EDPJN  [44] | Mozambique | 2006  2007 | 0.20  (0.07-0.43)  0.01  (0.03-0.27) | 13.7 (8.0-22.9) | 32.0  (22.3-44.6) | High |
| Papua New Guinea | RAJDQ  [35] | Madang | 2007 | 0.27  (0.07-0.57) | 9.1  (1.3-49.2) | 9.1  (1.3-49.2) | High |
| Papua New Guinea | UANQM  [33] | Madang | 2005 | 0.27  (0.10-0.57) | 17.1  (10.6-26.8) | 34.5  (23.7-48.5) | High |
| Tanzania | EDPJN  [44] | Tanzania | 2006  2007 | 0.07  (0.03-0.13)  0.07  (0.30-0.13) | 2.5  (1.1-5.4) | 11.5  (7.4-17.5) | High |
| Tanzania | GZQDA [32] / KGHRT [32] | Fukayosi | 2008 | 0.03  (0.00-0.13) | 19.9  (15.4-25.4) | 44.5  (38.4-51.1) | High |
| Tanzania | GZQDA [32] / KGHRT [32] | Yombo | 2008 | 0.1  (0.03-0.23) | 17.4  (11.2-26.5) | 25.7  (18.1-35.6) | High |
| Tanzania | GZQDA [32]/ KGHRT [32] | Fukayosi | 2007 | 0.1  (0.03-0.23) | 25.2  (18.0-34.5) | 39.7  (31.0-49.6) | High |
| Tanzania | GZQDA [32]/ KGHRT [32] | Yombo | 2007 | 0.20  (0.07-0.40) | 18.2  (9.1-34.4) | 41.9  (28.2-59.1) | High |
| Tanzania | UHUBT  [42] | Kilombero | 2008 | 0.00  (0.00-0.03) | 2.4  (0.8-7.4) | 5.0  (2.3-10.8) | Moderate |
| Tanzania | XXFCZ  [31] | Kibaha | 2007 | 0.07  (0.00-0.13) | 36.3  (30.5-42.9) | 54.9  (48.6-61.4) | High |
| Thailand | FMNNB  [36] | SMRU | 1996 | 0.10  (0.03-0.23) | 7.5  (4.8-11.7) | 8.6  (5.6-13.2) | Low |
| Thailand | KGJRP  [27] | SMRU | 1996 | 0.03  (0.00-0.13) | 2.6  (1.0-6.8) | 5.2  (1.7-14.7) | Low |
| Thailand | RGPFA  [7] | Bangkok | 1996 | 0.00  (0.00-0.03) | 1.2  (0.2-8.2) | No data | Very Low |
| Thailand | SAUSX  [11,30] | SMRU | 1998 | 0.00  (0.00-0.03) | 0.9  (0.1-6.4) | 0.9  (0.1-6.4) | Low |
| Thailand | SAUSX  [11,30] | Bangkok | 1998 | 0.10  (0.03-0.20) | No data | No data | Very Low |
| Thailand | USGDC  [40] | SMRU | 2002 | 0.00  (0.00-0.00) | 0.0  (.-.) | 0.0  (.-.) | Very Low |
| Uganda | CCEPC  [37] | Mbarara | 2003 | 0.27  (0.07-0.60) | 3.4  (2.4-4.8) | No data | Moderate |
| Uganda | DBCXT  [5,29] | Kampala | 2008 | 0.17  (0.07-0.37) | 4.6  (3.1-6.7) | No data | Low |

^1^MAP = Malaria Atlas Project; Median and 50% credible interval (25^th^-75^th^ centile) of the estimates (a posterior distribution) of *Plasmodium falciparum* endemicity are presented
^2^Evaluated in all patient with outcome, including those not participating in the pharmacokinetic component of the study; the worst case scenario is presented, i.e. for patients with no PCR available the new infection was assumed.
^3^ TIA: Transmission Intensity Area
