# Supplementary material for: Artemether-lumefantrine treatment of uncomplicated Plasmodium falciparum malaria: a systematic review and meta-analysis of day 7 lumefantrine concentrations and therapeutic response using individual patient data
Source: BMC Med. 2015 Sep 18;13:227. doi: 10.1186/s12916-015-0456-7 (PMC4574542; doi:10.1186/s12916-015-0456-7)
Supplement: Additional file 9: Table S4. — Studies with lumefantrine day 7 concentration measured, but not included in the pooled analysis. Only data for non-pregnant malaria patients with measured concentration on day 7 are presented. (DOCX 26 kb) [file 12916_2015_456_MOESM9_ESM.docx]

Supplementary Table 4. Studies with lumefantrine day 7 concentration measured not included in the pooled analysis. Only data for non-pregnant malaria patients, with measured concentration on day 7 are presented.

| Reference | Country | Study Year | Regimen | Number of patients with | | Day-7 Concentration  summary (ng/ml) | Comment |
| --- | --- | --- | --- | --- | --- | --- | --- |
|  |  |  |  | Day-7 concentration | Recrudescences |  |  |
| [65] | Thailand | 1998 | 4 doses over 3 days  3 doses over 2 days | Not stated  (out of 39 patients) | 9 | Not given | Requested data |
| [64] | Laos | 2003 | 6 doses over 3 days | 53 | 3 | Median (Range)  460 (135 – 2429) | Requested data |
| [66] | Europe and Columbia | Not given | 6 doses over 3 days | 15 | 0 | Not given | Requested data |
| [10] | Bangladesh | 2006-2007 | 6 doses over 3 days | 320 | 1 | Median (Range)  Non-DOT:  670.8 (0 – 4892.2  DOT:  860.3 (53.8 -6215.0) | Included in WWARN repository in 2015 after the analysis cut-off date |
| [13] | Tanzania | 2012 | 6 doses over 3 days | 20 | 1 | Mean (95% CI)  1,261 (999 to 1,522) | Published after the analysis cut-off date |

DOT: Directly Observed Therapy
